# Walking as a Window to the Brain: Redefining Gait in Neurology

**DOI:** 10.3390/medsci14030338

**Published:** 2026-06-23

**Authors:** Emmanuel Ortega-Robles, Mario Treviño, Elías Manjarrez, Oscar Arias-Carrión

**Affiliations:** 1 División de Neurociencias Clínica, Instituto Nacional de Rehabilitación Luis Guillermo Ibarra Ibarra, Mexico City 14389, Mexico; edortegar@gmail.com; 2 Laboratorio de Plasticidad Cortical y Aprendizaje Perceptual, Instituto de Neurociencias, Universidad de Guadalajara, Guadalajara 44130, Mexico; mario.trevino@academicos.udg.mx; 3 Instituto de Fisiología, Benemérita Universidad Autónoma de Puebla, Puebla 72570, Mexico; 4 Escuela de Medicina y Ciencias de la Salud, Tecnologico de Monterrey, Mexico City 14380, Mexico

**Keywords:** gait analysis, digital biomarkers, neurological disorders, mobility outcomes, precision neurology, translational neuroscience

## Abstract

Walking is not merely locomotion but a window into the nervous system, integrating cortical, subcortical, cerebellar, spinal, and peripheral networks into a unified motor behavior. Across neurological diseases—including Parkinson’s disease, atypical parkinsonism, cerebellar ataxias, stroke, multiple sclerosis, neuropathies, neuromuscular disorders, and functional gait syndromes—gait disturbances are among the most disabling clinical features, contributing to falls, loss of independence, institutionalization, and premature mortality. Traditional bedside observation remains indispensable, but it lacks the sensitivity and reproducibility needed to capture subtle, episodic, or prodromal abnormalities. Over the past decade, advances in wearable sensors, marker-based and markerless motion capture, pressure-sensitive walkways, force plates, artificial intelligence, and machine learning have positioned digital mobility outcomes as promising, ecologically valid biomarkers of neurological function. These measures can support differential diagnosis, provide prognostic information on falls and survival, and serve as sensitive endpoints in therapeutic trials. They may also detect early abnormalities, such as increased stride-to-stride variability or prolonged double-support time, before overt clinical deterioration becomes evident. Clinical applications are increasingly evident across disorders, including distinguishing Parkinson’s disease from atypical parkinsonism, quantifying treatment response in normal-pressure hydrocephalus, tracking progression in ataxia and multiple sclerosis, predicting functional decline in motor neuron disease, and guiding rehabilitation after stroke. Integration with neuroimaging, electrophysiology, and molecular biomarkers is beginning to reveal the circuits underlying variability, instability, and freezing, positioning gait as a systems-level marker of neural integrity. Nevertheless, methodological heterogeneity, limited disease-specific validation, insufficient longitudinal data, and lack of consensus on clinically meaningful parameters continue to constrain translation. Cognitive, affective, and environmental influences also remain insufficiently represented in digital frameworks, while equity, accessibility, algorithmic bias, and privacy require careful ethical governance. Reconceptualizing gait as a “sixth vital sign” reframes mobility as a multidimensional biomarker of neural and systemic health. With harmonized protocols, robust validation, multimodal integration, and appropriate ethical frameworks, gait analysis could become a cornerstone of precision neurology.

## 1. Introduction

Walking constitutes a complex motor behavior that reflects the integrated output of cortical, subcortical, cerebellar, and peripheral neural circuits. Each step embodies the orchestration of cortical planning, basal ganglia modulation, cerebellar coordination, spinal pattern generation, and continuous peripheral feedback [1,2,3]. This choreography, repeated thousands of times each day, renders gait not only a motor behavior but also a multidimensional biomarker of neural and systemic health [4].

With the rapid aging of populations worldwide, disorders that compromise mobility—including Parkinson’s disease (PD), stroke, dementia, cerebellar ataxias, multiple sclerosis, neuropathies, and neuromuscular diseases—are projected to rise sharply, creating profound challenges for healthcare systems and societies [5,6]. Gait and balance impairments are among the most disabling manifestations of these conditions, driving falls, institutionalization, dependence, and premature mortality [7,8]. Importantly, these impairments are not ancillary complaints: they are harbingers of progression and powerful predictors of long-term outcomes. In PD, for instance, dopaminergic therapy alleviates tremor and rigidity but often fails to restore locomotor stability, particularly in advanced stages where freezing of gait and postural instability dominate [9,10]. Similar therapeutic gaps persist in stroke and dementia, where cumulative disability from gait dysfunction continues to outpace advances in pharmacological and rehabilitative care [11].

For over a century, the neurological examination of gait—descriptions of shuffling, spastic, or ataxic patterns—has been a cornerstone of clinical practice. Nevertheless, bedside observation, indispensable though it remains, is limited by inter-rater variability and insensitivity to subtle, prodromal, or episodic abnormalities. Today, quantitative gait analysis offers a transformative alternative. For instance, dual-task gait is a sensitive marker for detecting prodromal cognitive Parkinson’s and Alzheimer’s diseases [12] or mild cognitive impairment [13].

By providing objective, reproducible, and multidimensional measures, it can distinguish overlapping syndromes, track progression, and predict outcomes with unprecedented precision [14,15,16]. Over the past decade, a technological convergence has driven this transformation: wearable inertial sensors, motion capture systems, pressure-sensitive walkways, and mobile health applications, amplified by artificial intelligence and machine learning, have enabled not only early detection but also continuous monitoring in real-world settings [17]. The integration of gait metrics with neuroimaging, electrophysiology, and molecular biomarkers is beginning to reveal the mechanistic circuits underlying instability, variability, and freezing [18,19].

Several key terms used throughout this review require clarification. Digital Mobility Outcomes (DMOs) refer to objective measures of mobility derived from digital health technologies, including spatiotemporal, kinematic, and dynamic features of gait captured in supervised or free-living conditions. Digital biomarkers are digitally measured characteristics, or sets of characteristics, which indicate normal biological processes, pathological processes, or responses to an intervention. Markerless motion capture refers to video-based movement analysis that estimates body landmarks and joint kinematics from standard camera recordings without the need for reflective markers, typically using computer vision and deep learning-based pose-estimation algorithms. Ecological validity refers to the extent to which a measurement captures behavior as it occurs in real-world contexts rather than under artificial or highly constrained laboratory conditions.

The pathophysiological framework for gait disturbances in basal ganglia disease has been substantially clarified at the circuit level. Takakusaki et al. proposed that normal gait depends on the integration of two interacting systems: a basal ganglia–brainstem system that regulates postural muscle tone and locomotor rhythm through reticulospinal pathways, and a cortical–basal ganglia loop that modulates volitional gait control. Disruption of basal ganglia output, as occurs in PD, impairs both automatic and intentional locomotor processes, providing a mechanistic account for why neither dopaminergic nor non-dopaminergic therapies fully restore gait in advanced disease [20].

This convergence underscores a profound conceptual shift. Gait should no longer be regarded as a peripheral symptom but rather as a higher-order function—an integrative marker of neural integrity. Emerging frameworks that distinguish continuous from episodic disturbances, or that map variability and stability signatures onto specific circuits, exemplify this reframing [6,21]. Reconceptualizing gait as a “sixth vital sign” of brain health positions walking not merely as movement but as a dynamic biomarker of neurological status across the lifespan [8,22].

This narrative review addresses a specific gap in the literature. Although previous reviews have examined neurological gait disorders, digital mobility outcomes, or specific measurement technologies, few have integrated the neural circuit foundations of gait with quantitative digital technologies across a broad spectrum of neurological conditions. In addition, the translational, regulatory, artificial intelligence, and ethical barriers that stand between research promise and clinical adoption have often been discussed separately rather than within a unified framework. Unlike reviews focused predominantly on a single technology, disease, or analytical domain, our approach is integrative and transdiagnostic, encompassing PD, atypical parkinsonism, cerebellar ataxias, multiple sclerosis, stroke, neuromuscular disorders, peripheral neuropathies, and functional gait syndromes. The review is organized around four conceptual domains: (1) the neurobiological foundations of locomotion; (2) the methodological landscape of quantitative gait analysis; (3) disease-specific gait signatures and their diagnostic, prognostic, and therapeutic relevance; and (4) the translational frontier, including methodological heterogeneity, regulatory readiness, artificial intelligence, and ethical governance. We argue that quantitative gait analysis, when implemented with validated digital tools, provides objective and ecologically valid measures of neural circuit function that complement existing clinical biomarkers across neurological diseases.

### Literature Search Strategy

This narrative review was prepared in accordance with the principles of the SANRA framework for the transparent reporting of narrative review articles. A structured literature search was conducted in PubMed/MEDLINE, Embase, Web of Science, and Scopus for publications indexed between January 1994 and March 2026. Search terms combined controlled vocabulary, when available, and free-text terms related to gait analysis, walking, locomotion, digital biomarkers, digital mobility outcomes, wearable sensors, inertial measurement units, motion capture, machine learning, artificial intelligence, Parkinson’s disease, atypical parkinsonism, cerebellar ataxia, multiple sclerosis, stroke, neuromuscular disorders, peripheral neuropathies, functional gait disorders, rehabilitation, and precision neurology. Eligible sources included peer-reviewed original studies, systematic reviews, meta-analyses, consensus statements, and clinical practice guidelines published in English and addressing quantitative gait measures, digital mobility outcomes, or clinically relevant gait phenotypes in neurological conditions. Studies were excluded when they focused exclusively on non-neurological musculoskeletal conditions, pediatric populations outside the scope of the review, animal models, purely technical engineering development without clinical validation, or non-peer-reviewed material. Landmark studies were selected based on methodological rigor, citation impact, representativeness of major conceptual or technological advances, and consensus among the authors, with priority given to recent evidence and studies explicitly addressing translational relevance, regulatory validation, artificial intelligence, ethical governance, or clinical implementation.

## 2. Human Gait: Neurobiological Foundations and Clinical Translation

### 2.1. Neural Control and Postural Stability

**Figure 1:**
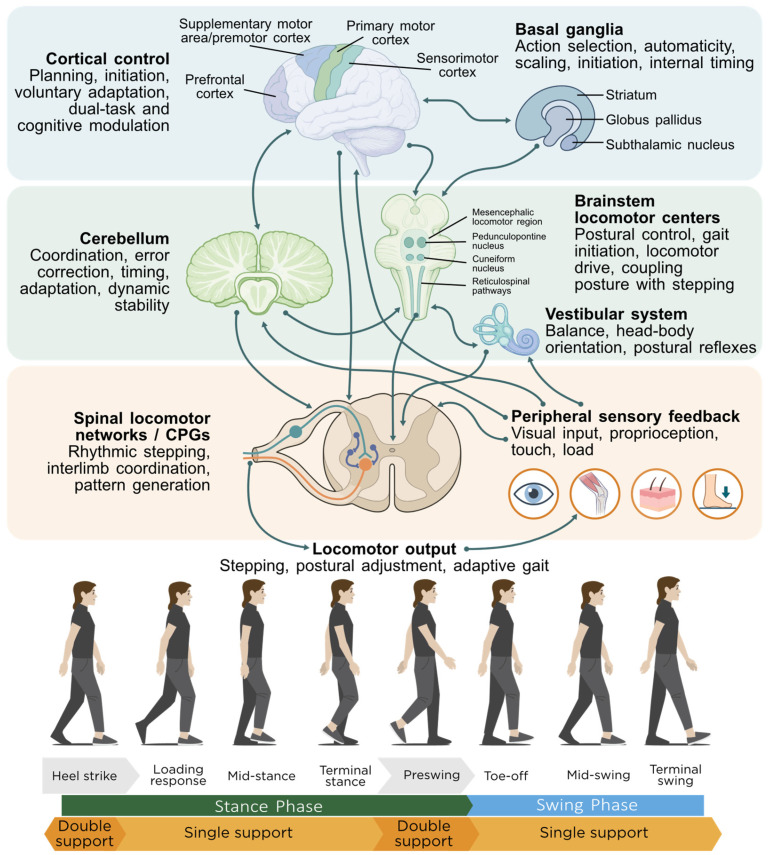
Neural control of gait and gait-cycle organization. The upper panel summarizes the distributed neural control of gait, including cortical planning, basal ganglia modulation, cerebellar coordination, brainstem and vestibular contributions, spinal locomotor networks, and peripheral sensory feedback. The lower panel illustrates the sequential phases of the gait cycle from heel strike to terminal swing, distinguishing stance and swing phases and the alternation between double- and single-support periods.

Human walking is often perceived as an automatic behavior, yet it depends on one of the most complex motor control systems in the human body [23]. Locomotion requires the coordinated activity of distributed neural networks that integrate motor planning, sensory feedback, and postural control [24]. Several regions of the central nervous system contribute to this process. The motor cortex initiates voluntary movement; the basal ganglia regulate movement amplitude and initiation; the cerebellum refines timing and coordination; and brainstem locomotor centers interact with spinal central pattern generators that produce the rhythmic activation of limb muscles necessary for walking (Figure 1) [20,24].

Equally important is the continuous flow of sensory information that allows the nervous system to adjust locomotion to environmental conditions [25]. Visual input provides information about obstacles and direction, vestibular signals contribute to equilibrium, and proprioceptive feedback from muscles and joints informs the brain about limb position and loading [26]. Together, these sensory systems allow walking to remain stable and adaptive even when the environment changes [27].

Walking can therefore be conceptualized as a controlled sequence of forward shifts in the body’s center of mass relative to the base of support provided by the feet [28,29]. During locomotion, the nervous system must constantly ensure that the center of mass remains within a stable region to prevent loss of balance [30]. Two related forms of stability help describe this process. Static stability refers to the ability to maintain an upright posture during quiet standing, typically assessed by measuring center-of-pressure sway beneath the feet [31]. Dynamic stability, by contrast, reflects the ability to maintain balance while the body is moving, particularly during the forward progression that characterizes walking [31].

Disruption of these stabilizing systems is a major contributor to falls in neurological disease [31]. Patients with PD, cerebellar disorders, multiple sclerosis, or stroke frequently exhibit delayed postural responses [32], abnormal integration of sensory information, and impaired dynamic balance control [33,34]. These abnormalities strongly correlate with fall risk and reduced functional independence [35,36]. For clinicians, understanding the neural foundations of balance is therefore essential for interpreting gait abnormalities and designing effective rehabilitation strategies.

An important refinement of our understanding of postural control is that it is not simply reflexive but involves anticipatory and predictive mechanisms. Horak proposed that postural orientation and postural equilibrium are the two fundamental functional goals of postural behavior and that they depend on the dynamic integration and reweighting of visual, vestibular, and somatosensory inputs according to task context. In neurological disease, the disruption of these integrative mechanisms, rather than the loss of individual sensory modalities per se, is a primary driver of postural instability and falls [27].

### 2.2. The Vestibular System and Gait in Neurological Disorders

The vestibular system plays a fundamental but frequently underappreciated role in the control of gait and dynamic postural stability. Through its peripheral receptors, the semicircular canals and otolith organs, it continuously signals head acceleration and spatial orientation to the brainstem, cerebellum, and cortex, enabling real-time corrective adjustments of muscle tone, trunk alignment, and locomotor trajectory during walking [37]. When this system is compromised, whether by primary vestibular pathology or as a consequence of a broader neurological disorder, the resulting gait disturbance reflects the loss of this continuous sensory feedback. In bilateral vestibulopathy, for example, patients characteristically exhibit increased stride variability, a widened base of support, and a marked deterioration of gait stability at slower walking speeds and under conditions of sensory conflict, a pattern that is quantitatively distinct from the gait signatures of cerebellar ataxia or functional gait disorders [37,38].

Vestibular dysfunction is also increasingly recognized as a clinically relevant but underdiagnosed contributor to gait impairment in common neurological conditions. In PD, deficient vestibular processing has been linked to freezing-of-gait episodes and abnormal postural responses, with neuroimaging evidence suggesting that disrupted vestibular-basal ganglia connectivity may partly underlie these phenomena [39,40]. In post-stroke patients, impaired central vestibular processing contributes to asymmetric weight-bearing, lateral trunk instability, and increased fall risk, and vestibular rehabilitation therapy has been shown in a systematic review and meta-analysis of randomized controlled trials to significantly improve both balance and gait outcomes in this population [41]. Across neurological conditions, the instrumented assessment of vestibular-related gait parameters, including stride variability, head-trunk coordination, and responses to sensory perturbations, offers a sensitive window into the integrity of vestibulospinal pathways and can assist both in differential diagnosis and in the objective monitoring of disease progression [42].

### 2.3. The Gait Cycle: Biomechanical Organization of Locomotion

At the mechanical level, locomotion follows a repeating pattern known as the gait cycle, which represents the fundamental unit of walking [43]. The cycle begins when the heel of one foot contacts the ground and ends when the same heel contacts the ground again during the next step [29]. This repeating sequence allows the body to move forward while maintaining stability.

The gait cycle is classically divided into two principal phases. During the stance phase, which accounts for approximately 60% of the cycle, the foot remains in contact with the ground and supports the body’s weight. This phase includes the initial heel contact, the period when the body moves over the supporting foot, and the final push-off that propels the body forward. The swing phase, which accounts for the remaining 40% of the cycle, occurs when the foot lifts from the ground and moves forward in preparation for the next step [7,44,45].

Although these proportions remain relatively stable in healthy adults, subtle alterations in the gait cycle’s structure can reveal underlying neurological dysfunction. In PD, for example, patients frequently demonstrate shortened steps, diminished heel strike, and reduced foot clearance [10,15,46]. These abnormalities reflect bradykinesia, a reduction in movement speed and amplitude that arises from dysfunction of dopaminergic circuits within the basal ganglia [9,47,48]. Quantitative measurement of stance and swing phases has therefore become an important tool in clinical neurology, allowing clinicians to monitor disease progression and evaluate therapeutic responses to pharmacological treatment, neuromodulation, and rehabilitation interventions [49].

Normative reference values for gait cycle parameters vary with age, sex, and walking speed. In healthy older adults, stance duration typically ranges from 60 to 64% of the gait cycle, with a systematic prolongation of double-support time and a reduction in walking speed with advancing age [50]. These normative data provide the essential baseline against which disease-related deviations can be identified and quantified, reinforcing the value of age-stratified reference ranges in clinical gait analysis [50].

**Figure 2:**
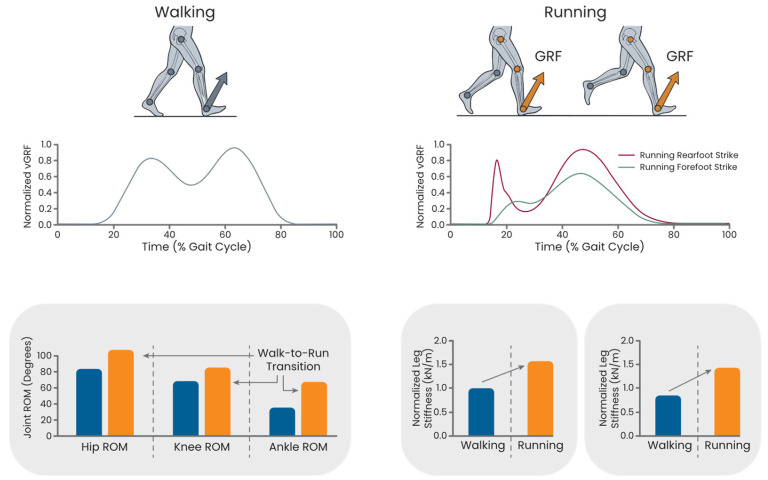
Biomechanical differences between walking and running. (**Top**) sagittal-plane illustrations of lower-limb configuration and ground reaction force (GRF) vectors during walking and running. (**Middle**) representative vertical GRF profiles, showing the double-hump pattern of walking and the distinct loading profiles associated with rearfoot and forefoot running strikes. (**Bottom**) summary of changes in joint range of motion and normalized leg stiffness across the walk-to-run transition. GRF, ground reaction force; ROM, range of motion; vGRF, vertical ground reaction force.

The distinction between walking and running adds a further layer. These two gaits are not simply slow and fast versions of the same motor program: they differ in foot-strike mechanics, force transmission, joint kinematics, and muscle coordination, with direct consequences for injury risk, motor-control modeling, and clinical assessment. Walkers use a consistent heel-to-toe strike with peak plantar pressures under the heel, whereas runners recruit a broader range of strategies: rearfoot, midfoot, and forefoot. During running, rearfoot striking produces a distinct impact peak in the vertical ground reaction force (vGRF) and steeper loading rates, while forefoot striking attenuates or eliminates this transient [51,52,53]. Running also increases the range of motion at the hip, knee, and ankle, especially in sagittal and transverse planes [54,55,56,57]. Six-degree-of-freedom knee analyses reveal that, across the walk-to-run transition, flexion–extension range of motion and axial tibial rotation increase non-linearly, accompanied by greater proximal–distal and mediolateral tibial translations [55,56]. Global leg and joint stiffness rise with speed, a regulation thought to manage higher peak forces; compliant surfaces paradoxically increase stiffness further, particularly at the knee and ankle (Figure 2) [58].

**Figure 3:**
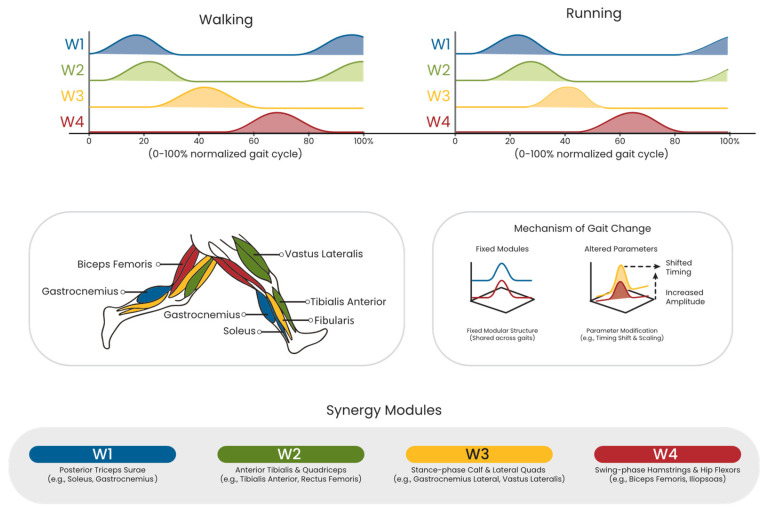
Muscle synergy modules shared across walking and running. The upper panels show four representative muscle synergy modules (W1–W4) across the normalized gait cycle during walking and running. The lower-left panel maps the main lower-limb muscle groups contributing to each module, and the lower-right panel illustrates how changes in timing and amplitude can modify shared synergy structures across gait modes [63]. W1–W4, muscle synergy modules.

Despite these mechanical differences, walking and running appear to share a common set of muscle synergies, low-dimensional activation modules that spinal and supraspinal circuitry uses to coordinate dozens of muscles simultaneously. The key difference is temporal: one stance-related synergy module shifts earlier in the gait cycle during running to match the shorter stance phase [59,60]. Global lower-limb co-activation and center-of-mass displacement increase across the walk–run transition, reflecting a switch toward whole-limb stiffness regulation [61]. Foot-strike pattern modifies synergy weights and timing, particularly the tibialis anterior contribution, without fundamentally restructuring the modules themselves [62], consistent with simulation work showing that substantial biomechanical changes can be produced by tuning synergy parameters rather than rebuilding modular architecture [63]. Running also exhibits higher approximate entropy in hip and knee kinematics than walking, suggesting the locomotor system increases movement complexity as a strategy to distribute loads across a wider kinematic space [64]. This connects directly to the variability discussion: at higher speeds, a controlled increase in stride-to-stride variability may be protective rather than pathological (Figure 3).

### 2.4. Spatial–Temporal Organization: The Rhythm of Walking

Beyond the structural organization of the gait cycle, walking can be characterized by spatial and temporal parameters that describe the rhythm and efficiency of locomotion [65]. These parameters include walking velocity, stride length, step length, cadence, and stride-to-stride variability [66]. Together, they provide a detailed description of how efficiently an individual moves through space.

In healthy individuals, these parameters remain highly consistent, reflecting stable neural control of locomotion and efficient biomechanical coordination [16,67]. The nervous system maintains this rhythmic consistency even as it adapts to variations in terrain or the direction of movement [68]. When neurological disease affects locomotor circuits, however, this rhythmic organization often deteriorates [69,70].

Patients with cerebellar ataxia, for instance, frequently display irregular step timing and inconsistent step lengths, reflecting impaired cerebellar contributions to motor coordination and timing [71]. Individuals with balance impairment often increase the duration of double support, the phase of the gait cycle during which both feet remain on the ground simultaneously. This strategy increases stability but slows forward progression [72].

Stride variability has emerged as a particularly informative measure of neurological dysfunction. Increased variability in step timing or length has been associated with heightened fall risk and may also reflect impaired cognitive control of walking [73,74]. These findings have reinforced the idea that locomotion is not purely automatic but requires the participation of higher-order cognitive processes such as attention and executive control [10,75].

**Figure 4:**
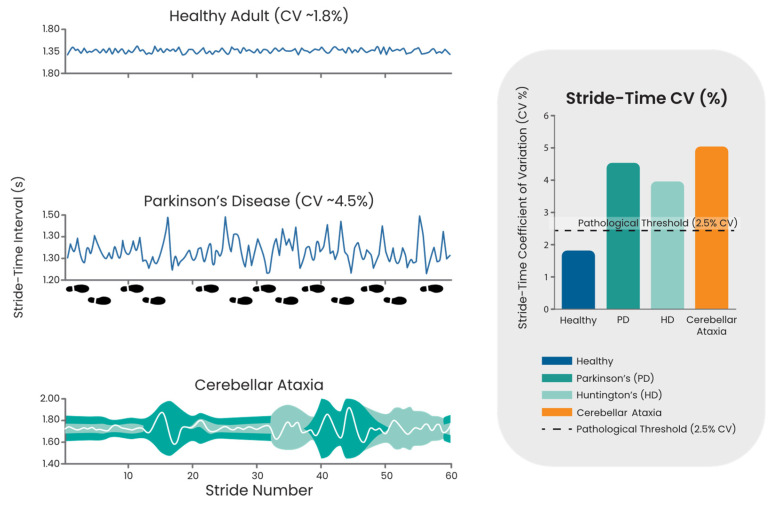
Stride-to-stride variability as a marker of impaired locomotor control. Representative stride-time traces show low temporal variability in healthy adults, increased irregularity in Parkinson’s disease, and larger fluctuations in cerebellar ataxia. The bar chart summarizes the stride-time coefficient of variation values across healthy adults and selected neurological disorders, with the dashed line indicating an approximate pathological reference threshold. CV, coefficient of variation; HD, Huntington’s disease; PD, Parkinson’s disease.

Every step a person takes differs slightly from the last. These stride-to-stride fluctuations in timing and spatial scaling are not measurement noise; they reflect how the nervous system generates, monitors, and corrects the locomotor pattern in real time. Unlike mean gait parameters such as average speed or stride length, variability captures the consistency of motor output and, by extension, the integrity of the circuits that produce it. Temporal variability, chiefly the coefficient of variation (CV) of stride time, is tightly linked to inter-limb coordination and rhythmogenesis. After a stroke, increased stride-time variability accounts for a substantial share of coordination deficits even after controlling for walking speed, whereas stride-length variability does not [76,77]. This dissociation implies that the temporal and spatial components of the stride cycle are regulated, at least in part, by separable control loops. In basal ganglia disorders, the temporal component is especially affected: patients with PD and Huntington’s disease exhibit stride-time variability two to three times that of age-matched controls, even when mean walking speed is near normal (Figure 4) [78,79].

Spatial variability, meanwhile, dominates the clinical picture in cerebellar ataxia, where step-to-step spatial fluctuations during free-living walking track ataxia severity with enough resolution to detect small within-stage disease changes [80]. Meta-analyses and large cohorts confirm that variability metrics, particularly stride-time CV, discriminate pathological from healthy gait better than mean speed or stride length, with pathological thresholds clustering around 2.3–2.6% CV [81,82,83].

In Huntington’s disease, variability measures yield larger effect sizes than mean parameters and correlate more strongly with motor and functional clinical scales [79], while in Alzheimer’s disease (AD), elevated stride variability marks cortical-cognitive involvement and helps distinguish AD from other neurodegenerative conditions [81]. A critical nuance is that increased variability does not always indicate impaired locomotor control. In healthy older adults, stride-to-stride fluctuations may increase with age [78,79], potentially reflecting greater neuromuscular noise rather than a fundamental change in the underlying control strategy [84]. Interpreting variability, therefore, requires complementary measures, nonlinear dynamics, perturbation responses, or dual-task paradigms, to separate noise from genuine control deficits.

Beyond conventional linear variability metrics, nonlinear analytical approaches capture higher-order properties of locomotor control that may not be detected by mean values, standard deviations, or coefficients of variation. Fractal dynamics, commonly quantified using detrended fluctuation analysis, describe the long-range temporal correlations present in stride-to-stride fluctuations. In healthy walking, stride intervals show structured correlations across time, reflecting a balance between automatic rhythmicity and flexible adaptation to internal and environmental demands. In neurological disease, disruption of these correlations may indicate degradation of automatic locomotor control, impaired cognitive-motor integration, or altered basal ganglia, cerebellar, and spinal contributions to gait regulation [16]. Entropy-based measures, including approximate entropy and sample entropy, provide complementary information by quantifying the regularity and predictability of gait signals. Reduced entropy may reflect excessive stereotypy and loss of adaptive flexibility, whereas excessive irregularity may reflect unstable or poorly coordinated locomotor output, depending on the disorder, signal analyzed, and walking condition. Multiscale entropy extends this approach by evaluating signal complexity across multiple temporal scales, which may help distinguish disorders that show similar levels of linear variability but differ in the organization of their locomotor dynamics. These nonlinear metrics are therefore increasingly relevant as digital biomarkers because they capture aspects of neurological gait dysfunction that stride-time coefficient of variation, stride length, or walking speed alone may not resolve, including loss of automaticity, impaired adaptability, fall risk, and progression of neurodegenerative disease [16,85].

Technological advances have significantly expanded the clinical importance of these measurements. Wearable sensors and smartphone-based monitoring systems now enable continuous recording of spatial and temporal gait parameters in everyday environments [86]. As a result, these parameters are increasingly viewed as digital biomarkers that can detect early mobility impairment and monitor disease progression in neurological disorders [36].

The clinical interpretation of spatial-temporal gait parameters is facilitated by organizing them into distinct domains. Factorial analyses of gait in older adults and neurological populations consistently yield five semi-independent domains: pace (gait speed, stride length), rhythm (cadence, stride time), variability (stride-to-stride fluctuations), postural control (step width, base of support), and phase (stance and swing proportions) [50,87]. This domain structure is clinically meaningful: pace parameters are most sensitive to disease severity and pharmacological response, whereas variability parameters are more closely associated with cognitive function, fall risk, and non-dopaminergic dysfunction [87]. Recognizing these distinctions guides both the selection of outcome measures in clinical trials and the interpretation of gait findings in individual patients (Table 1).

### 2.5. Kinematics: The Geometry of Movement

While spatial and temporal parameters describe the rhythm of walking, kinematics focuses on the geometry of movement by analyzing how different body segments move through space during locomotion [88,89]. In gait analysis, kinematic measurements capture the angles and trajectories of major joints such as the hip, knee, and ankle throughout the gait cycle.

In healthy locomotion, these joint movements follow smooth and coordinated trajectories that allow efficient forward progression while maintaining balance [29]. The hip extends as the body moves over the supporting leg, the knee flexes during the swing phase to allow the foot to clear the ground, and the ankle contributes to push-off during the late stance.

Neurological and musculoskeletal disorders can distort these trajectories in distinct ways. Degenerative joint disease may reduce the range of motion available at affected joints [89]. Spasticity following a stroke may produce rigid or exaggerated movement patterns [90,91]. PD often results in reduced limb movement amplitude, including diminished arm swing and shortened step length [92].

Patients frequently adopt compensatory strategies to maintain mobility. A common example is hip circumduction, in which the leg moves outward in a circular motion to prevent the foot from dragging on the ground during the swing phase in hip osteoarthritis [93] or in hemiplegia [94].

Historically, detailed kinematic analysis required specialized motion-capture laboratories equipped with optical tracking systems and reflective markers attached to the body [89,95]. Advances in wearable inertial sensors and computer vision technologies now allow joint trajectories to be measured using portable devices or video recordings [96]. These developments have expanded access to gait analysis and allow clinicians to evaluate locomotion in more natural environments [7,97].

To address the complexity of multi-joint kinematic data in clinical settings, composite summary indices have been developed. The Gait Profile Score (GPS) and Gait Deviation Index (GDI) integrate deviations across multiple joint angles relative to normative data into a single scalar measure of overall kinematic pathology, facilitating comparison across patients, conditions, and time points [98]. These indices are particularly valuable in neurological practice, where simultaneous abnormalities at the hip, knee, and ankle joints make interpretation of individual parameters challenging and where a global measure of kinematic deviation provides a concise indicator of treatment response.

### 2.6. Kinetics: The Forces That Sustain Locomotion

Kinematics describes how the body moves, whereas kinetics explains the forces that generate that movement [28]. Walking depends on continuous interaction between the body and the ground, producing ground reaction forces each time the foot contacts the surface. These forces act in three principal directions. Vertical forces support body weight, anteroposterior forces contribute to forward propulsion, and mediolateral forces help maintain balance. The coordinated regulation of these forces allows the body’s center of mass to move smoothly over the base of support during walking [29,52].

Neurological disorders can disrupt this delicate balance of forces. In PD, impaired motor scaling reduces propulsive force, contributing to the short, hurried steps known as festination and increasing the likelihood of freezing episodes during walking [49,99]. In neuromuscular disorders or degenerative joint disease, reduced muscle strength may limit propulsion, resulting in slower walking speed and rapid fatigue [89].

A clinically important extension of ground reaction force analysis is the computation of joint moments across the gait cycle. Joint moments at the hip, knee, and ankle quantify the specific muscular demands required to sustain locomotion and reveal how neurological or musculoskeletal pathology redistributes loading across joints [100]. In PD, for example, reduced ankle plantar flexor moments are accompanied by a compensatory increase in hip flexor moments [99]. Such joint-level kinetic data provide mechanistic targets for rehabilitation that go beyond the spatiotemporal parameters visible in clinical observation.

Traditionally, kinetic analysis requires laboratory-based force plates embedded within walkways. Recent technological advances have enabled wearable sensors to estimate locomotor forces during everyday movement [96,101]. These innovations enable gait to be studied outside laboratory settings and provide clinicians with valuable information about functional mobility and rehabilitation potential.

### 2.7. Electromyography: Mapping Neural Commands to Muscle Activity

Movement ultimately depends on the activation of skeletal muscles, which translate neural commands into mechanical force. Electromyography (EMG) provides a method for recording the electrical activity generated when motor neurons activate muscle fibers [102,103]. Surface electrodes placed on the skin can capture activity from superficial muscles, whereas intramuscular electrodes allow more precise recordings from deeper muscle groups. While walking, muscles activate in highly coordinated sequences that allow efficient propulsion and stable posture [103]. Agonist and antagonist muscle groups alternate in carefully timed patterns that minimize energy expenditure while maintaining balance.

An important organizational principle of EMG during walking is the concept of muscle synergies. Rather than controlling each muscle independently, the central nervous system combines muscles into a small number of co-activation modules, typically four or five, that account for most of the variance in multi-muscle EMG during the gait cycle [104]. These synergies represent stereotyped neural commands that are activated at specific phases of the gait cycle to provide propulsion, weight support, balance, and foot clearance. In neurological disorders, synergy structure is disrupted: patients with stroke, for example, show a reduced number of synergies and abnormal merging of normally distinct modules, reflecting loss of independent neural control of locomotor subtasks [103]. Synergy analysis thus provides a window into the organization of spinal and supraspinal locomotor circuits and may serve as a biomarker of neurological recovery.

Disruption of these patterns can reveal important information about neurological dysfunction. In dystonia, for example, inappropriate co-contraction of opposing muscles results in inefficient, unstable movement [105,106]. During muscle fatigue, the frequency characteristics of EMG signals change, reflecting declining muscle performance [107]. When EMG recordings are combined with kinematic and kinetic measurements, they provide a comprehensive picture of locomotion by linking neural activation patterns with biomechanical movement and force generation [102,108]. This integrated approach is increasingly used to guide rehabilitation strategies and optimize neuromodulation therapies, such as deep brain stimulation [9,109].

### 2.8. Digital Gait Biomarkers and the Emerging Paradigm Shift

For centuries, neurologists described gait primarily through clinical observation. Terms such as shuffling, spastic, or ataxic gait provided valuable descriptive insights but lacked the precision required for detailed mechanistic analysis [110]. Recent advances in wearable technologies, sensor-based monitoring, and machine learning are transforming gait analysis into a quantitative science [36,86]. Portable accelerometers, inertial measurement units, and smartphone-based systems can now record locomotor behavior continuously in real-world environments [17,111]. These technologies provide detailed information about gait dynamics that was previously accessible only in specialized research laboratories. For neuroscientists, quantitative gait analysis offers a powerful approach to studying how neural circuits generate and regulate locomotion. For clinicians, digital gait metrics provide objective biomarkers that can support earlier diagnosis, track disease progression, and evaluate the effectiveness of therapeutic interventions.

Emerging evidence suggests that subtle gait abnormalities may appear years before the onset of classic neurological symptoms in disorders such as PD [10,17,112,113,114]. Continuous monitoring of mobility, therefore, has the potential to provide early indicators of brain network dysfunction and may enable earlier intervention. Gait analysis is thus undergoing a profound transformation, evolving from a descriptive clinical art into a predictive and data-driven discipline [115].

The relationship between gait and cognitive health has emerged as one of the most clinically consequential discoveries in this field. Longitudinal studies have demonstrated that declining gait velocity predicts future cognitive impairment, dementia, and falls, often years before clinical symptoms are recognized [10,11]. This cognitive-motor link suggests that walking is not merely a motor task but an integrative readout of distributed brain network function. As a result, digital gait biomarkers may ultimately serve not only as markers of motor circuit integrity but also as sensitive, non-invasive windows into the health of cortical, subcortical, and cerebellar networks that support both cognition and mobility.

As digital mobility technologies become integrated into clinical practice, locomotor metrics may increasingly serve as a window into brain health itself, redefining how neurological diseases are detected, monitored, and ultimately treated [10,36].

## 3. Critical Appraisal of Gait Analysis Methods

### 3.1. Objective Gait Analysis: Promise and Persistent Challenges

The scientific study of gait has evolved substantially over the past two decades. Historically, clinicians relied primarily on bedside observation to identify abnormalities in walking patterns. Experienced neurologists could recognize characteristic gait phenotypes—such as parkinsonian shuffling, cerebellar ataxia, or spastic hemiparetic gait—through visual inspection alone [110]. While such observational skills remain essential in clinical practice, advances in technology have ushered in a new era of objective gait analysis, enabling quantitative measurement of locomotion with increasing precision. A wide range of analytical tools now enables the detailed assessment of gait kinematics, kinetics, spatial–temporal parameters, and muscle activation patterns [88]. These technologies offer important advantages. They provide objective measurements that can refine clinical diagnosis, detect early disease-related changes, and quantify responses to therapeutic interventions. In neurological disorders such as PD, multiple sclerosis, stroke, and cerebellar ataxia, gait metrics are increasingly used as sensitive markers of disease severity and functional impairment [86].

The clinical and scientific value of these tools ultimately depends on a practical methodological question: how accurately can gait be measured? Optical motion capture coupled with embedded force plates remains the gold standard for joint kinematics and kinetics but is confined to laboratories and prohibitively expensive for routine clinical use [67,116,117]. Nearly all wearable-validation studies use these systems as the reference comparator in both healthy and neurological cohorts [117,118,119,120]. Wearable inertial measurement units (IMUs) show good-to-moderate agreement with optical systems for joint kinematics; spatiotemporal parameters range from moderate to poor depending on the algorithm, sensor placement, and sampling rate [118,119]. Single- and multi-point IMUs are validated for speed, stride length, and cadence and are now used clinically in PD, stroke, multiple sclerosis, obesity, and osteoarthritis [118,121]. For running, IMUs are the most commonly used wearable devices; contact time, stride length and frequency, and tibial acceleration generally show good validity and reliability relative to lab standards, though accuracy depends strongly on placement and sampling rate [116,122].

**Figure 5:**
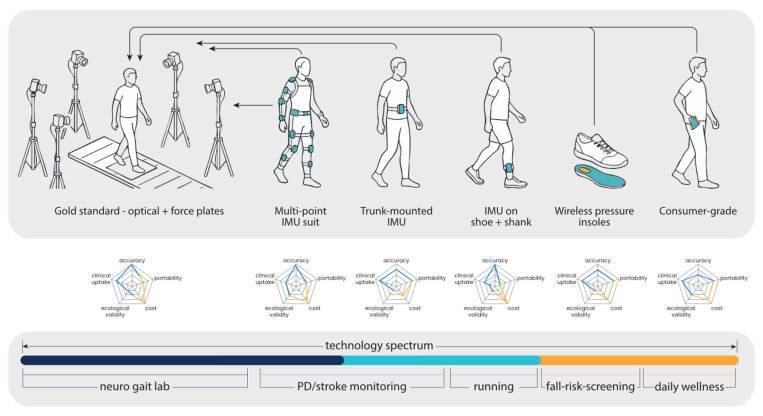
Gait measurement technologies: from laboratory systems to wearable and consumer sensors. Overview of gait analysis technologies across a continuum from optical motion capture with force plates to IMU-based systems, pressure insoles, and consumer-grade devices. Radar plots summarize relative trade-offs in accuracy, portability, cost, ecological validity, and clinical uptake. The lower spectrum indicates typical application contexts, from neuro-gait laboratories and disease monitoring to running assessment, fall-risk screening, and daily wellness tracking. IMU, inertial measurement unit; PD, Parkinson’s disease.

Pressure insoles complement IMUs by providing direct gait-event detection and plantar-pressure quantification. They show good timing accuracy relative to force plates for heel strike, toe-off, and stance duration [123,124]. Wireless insoles deliver reliable vGRF and center-of-pressure trajectories across walking, stairs, squats, and jumps, though absolute force magnitudes often differ from force plates; qualitative patterns and test–retest reliability are fair to excellent [117,120]. Multi-sensor systems (insoles, IMUs, and distance sensors) estimate speed, cadence, and stride length with excellent agreement (ICC > 0.95) against stereophotogrammetry across PD, multiple sclerosis, chronic obstructive pulmonary disease, heart failure, and hip fracture populations [125]. At the consumer end, smartphone accelerometers and commercial trunk sensors have been validated with agreement ranging from excellent to poor depending on parameter and cohort [119,121], while many commercial running-gait devices remain incompletely validated [122,126]. Despite growing clinical uptake, standardization of sensor protocols, placement conventions, and outcome definitions is still lacking (Figure 5) [67,116,127,128,129].

Despite these advances, interpreting gait analysis remains complex. Each methodological approach has distinct strengths and limitations that must be carefully considered when designing studies, interpreting results, or implementing gait analysis in routine clinical practice, as in the case of vision-based gait analysis systems [130]. In addition, the field continues to face significant challenges related to methodological heterogeneity. Different laboratories often employ distinct protocols, sensor configurations, and analytical algorithms, which can complicate comparisons across studies and limit reproducibility [131,132]. The absence of harmonized standards has therefore become a major barrier to broader clinical translation.

To address these challenges, there is growing recognition that the field requires consensus frameworks to establish robust, disease-specific protocols for gait measurement. Such frameworks would allow gait metrics to be compared reliably across research centers and clinical trials, thereby strengthening the role of gait analysis as a translational bridge between neuroscience, rehabilitation, and clinical neurology [7,36].

A related, clinically important distinction is between gait measured in supervised laboratory conditions and gait performed in daily life. Patients often demonstrate substantially better performance in clinical settings than at home, a discrepancy that reflects the gap between motor capacity and habitual performance [36]. Free-living gait monitoring using body-worn sensors captures how patients move in their natural environments, during multi-tasking, and in varied motivational contexts, thus revealing subtle motor impairments that may not manifest during brief standardized walk tests [114]. This ecological validity gap underscores the importance of developing ambulatory monitoring protocols that complement, rather than replace, laboratory-based gait assessments.

Before considering each technology in detail, Table 2 summarizes the main gait analysis devices, their measurement principles, strengths, and limitations.

### 3.2. Motion Capture Systems: From Laboratory Precision to Clinical Translation

Among the available methodologies, marker-based three-dimensional motion capture remains the reference standard for detailed biomechanical analysis of gait [133]. This approach relies on multiple infrared cameras that track reflective markers placed on carefully defined anatomical landmarks [134]. By reconstructing the spatial coordinates of these markers in three dimensions, motion capture systems generate precise measurements of joint angles, segmental trajectories, and limb movements throughout the gait cycle [134]. When combined with force measurements, they also allow the calculation of joint moments and mechanical power during walking.

The precision and reliability of marker-based motion capture have made it the gold standard in biomechanical research for several decades [135]. These systems have played a crucial role in establishing normative datasets for gait mechanics and in identifying biomechanical signatures associated with neurological and musculoskeletal disorders. However, the complexity of these systems also limits their clinical scalability. Motion capture laboratories require specialized equipment, trained personnel, and time-consuming preparation, including accurate marker placement and careful camera calibration [136]. Consequently, their use is largely confined to specialized research centers and advanced gait laboratories. Nevertheless, marker-based systems remain indispensable because they serve as benchmarks against which newer, more portable methods are validated.

In recent years, markerless motion capture has emerged as an important alternative. These systems use depth cameras or standard video recordings combined with advanced computer vision algorithms to estimate body posture and joint positions without the need for physical markers [137]. Advances in deep learning have significantly improved pose estimation accuracy, enabling these systems to approach the precision of traditional motion capture under controlled conditions [138].

Despite these advances, limitations remain. Measurements of joint motion in the frontal and transverse planes remain less reliable, particularly when movements are partially obscured or when lighting conditions vary [139]. Nevertheless, markerless technologies offer substantial advantages in terms of accessibility and scalability. They allow gait analysis to be performed in clinics, rehabilitation facilities, or even patients’ homes. Early clinical studies have already demonstrated their usefulness in monitoring motor responses to levodopa therapy in patients with PD [132]. Their translational value lies precisely in this ability to balance acceptable measurement accuracy with practical feasibility outside specialized biomechanics laboratories.

Taken together, the evolution of motion capture technologies reflects a broader shift in gait analysis from laboratory-bound precision towards clinically accessible measurement. Marker-based systems remain the gold standard against which all emerging technologies are benchmarked, having established the normative and biomechanical foundations of the field over several decades [140,141]. However, their inherent complexity and infrastructural demands limit their reach beyond specialized centers [136]. Markerless systems, driven by advances in deep learning and computer vision, now offer a viable path towards broader clinical deployment, achieving acceptable accuracy for spatiotemporal and sagittal plane kinematics while remaining constrained in the frontal and transverse planes [141,142]. Early applications in neurological populations, including the monitoring of motor responses to levodopa in PD, suggest that these technologies are beginning to bridge the gap between research-grade biomechanics and routine clinical practice [143,144]. As validation frameworks mature and accessibility improves, markerless motion capture is poised to become an integral component of the quantitative gait assessment toolkit in neurology.

### 3.3. Wearable Devices: Capturing Gait in Everyday Environments

Perhaps the most transformative development in contemporary gait analysis has been the emergence of wearable sensing technologies, such as IMUs [119]. The IMUs are miniaturized devices that integrate accelerometers, gyroscopes, and sometimes magnetometers, which can be attached to various parts of the body, including the trunk, wrists, and lower limbs [145,146]. These sensors record movement continuously and allow researchers to derive parameters such as stride length, cadence, angular velocity, and postural sway.

Unlike laboratory-based methods, wearable sensors capture mobility in naturalistic environments, providing insight into how neurological disorders affect everyday functioning. This ecological validity represents a major advantage [86]. Traditional laboratory assessments typically measure gait during brief, structured tasks that may not reflect real-world mobility. Wearable sensors, by contrast, can record locomotor behavior over days or even weeks, allowing clinicians to evaluate fluctuations in motor performance and to identify patterns of mobility impairment that may not be apparent during a single clinical visit [86].

Wearable technologies have already been applied in a wide range of neurological conditions. Studies have demonstrated their ability to detect gait abnormalities and monitor disease progression in PD, multiple sclerosis, cerebellar ataxia, stroke, healing of tibial fractures, and progressive supranuclear palsy [36,147]. In PD, for example, wearable sensors have been used to quantify freezing episodes, gait variability, and postural instability during daily activities.

Several validated wearable systems have reached sufficient clinical maturity to be deployed in routine neurological practice. In PD, the most extensively evaluated devices include the Mobility Lab system (APDM/Clario), which uses between one and six wireless Opal inertial sensors placed on the feet, lower back, sternum, and arms to quantify spatiotemporal gait parameters, arm swing, and postural transitions in both clinical and home settings [148]. The PDMonitor^®^ (PD Neurotechnology Ltd., London, UK, CE Class IIa) employs five inertial measurement unit (IMU) sensors worn simultaneously on both wrists, ankles, and waist to continuously capture gait parameters, freezing episodes, tremor, and bradykinesia over periods of up to seven days; its algorithms have been validated against neurologist Unified Parkinson’s Disease Rating Scale (UPDRS) ratings in multi-site clinical trials [149]. The Personal KinetiGraph^®^/PKG^®^ (Global Kinetics Pty Ltd./PKG Health, Melbourne, Australia) uses a single wrist-worn device to generate motor diaries quantifying dyskinesia and bradykinesia, while STAT-ON™ (Sense4Care S.L., Cornellà de Llobregat, Barcelona, Spain) and Kinesia 360™/KinesiaU™ (Great Lakes NeuroTechnologies Inc., Cleveland, OH, USA) provide complementary approaches using lumbar or upper-limb sensors, respectively. As of 2024, the United Kingdom National Institute for Health and Care Excellence (NICE) conditionally endorsed five of these technologies: PKG^®^, Kinesia 360™, KinesiaU™, PDMonitor™, and STAT-ON™. These are considered reliable options for remote monitoring to inform treatment decisions in PD [148]. While these systems demonstrate the maturity of wearable technology in movement disorders, equivalent validated platforms for other neurological conditions, such as multiple sclerosis, cerebellar ataxia, stroke, and fall risk, remain comparatively less developed, underscoring the need for broader disease-specific validation programs [150].

However, the rapid expansion of wearable technologies has also introduced methodological complexity. Studies differ widely in sensor placement, recording duration, and analytical algorithms used to derive gait parameters. Without careful standardization and validation against gold-standard laboratory methods, results may be difficult to compare across studies. For gait analysis using wearables to achieve full scientific credibility, harmonized protocols and disease-specific calibration will be essential.

### 3.4. Force Plates: Measuring the Mechanics of Balance and Propulsion

Force platforms represent a cornerstone of laboratory-based gait analysis [29,151,152]. These devices measure the forces generated when the foot interacts with the ground, allowing researchers to quantify ground reaction forces and the movement of the center of pressure beneath the feet [151,152]. These measurements provide detailed information about both static balance during standing and dynamic stability during walking.

When force plates are integrated with motion capture systems, they enable a comprehensive biomechanical analysis of locomotion [29,151]. Motion capture captures how body segments move through space, whereas force plates measure the mechanical forces that drive and stabilize these movements [151]. Together, these technologies allow researchers to understand not only the geometry of movement but also the underlying dynamics of locomotor control.

Force plate analysis has been particularly valuable in the study of neurological disorders associated with postural instability [152]. Characteristic patterns of ground reaction forces and center-of-pressure trajectories have been identified in conditions such as PD, multiple sclerosis, stroke, and cerebellar ataxia [36,147]. These measurements provide mechanistic insights into balance control and may help identify individuals at increased risk of falls.

Despite their advantages, force plates also present practical limitations. Measurements are highly sensitive to the specific hardware used, and different platforms may produce subtly different outputs [86,100]. For longitudinal studies and clinical trials, maintaining consistency in equipment and calibration is therefore critical. Nevertheless, force plates remain indispensable tools for high-precision analysis of locomotor biomechanics.

### 3.5. Pressure Sensors: Practical Tools for Spatial–Temporal Analysis

Instrumented walkways equipped with pressure-sensitive mats represent a more pragmatic approach to gait measurement for clinical and research purposes [153]. These systems have also been employed with young athletes to detect the timing and location of foot contacts during overground walking and to provide reliable measurements of spatial–temporal parameters, including step length, stride length, cadence, and walking speed [154]. Because they are relatively easy to operate and require minimal preparation time, pressure-sensitive walkways have gained widespread use in both clinical research and rehabilitation settings. Their reliability and practicality have led to increasing recognition by regulatory agencies as acceptable tools for mobility assessment in clinical studies [153,155].

A related innovation involves pressure-sensitive insoles, which embed miniature sensors within footwear. These devices measure foot–ground interactions during natural walking and enable gait data collection outside the laboratory [101,123]. By capturing locomotor behavior during everyday activities, pressure-sensing insoles provide valuable information about functional mobility.

However, these devices also have limitations. Compared with force plates, pressure sensors may underestimate vertical ground reaction forces and provide less accurate measurements of center-of-pressure trajectories. Consequently, their ability to quantify kinetic parameters is more limited [101]. Nevertheless, pressure-based technologies represent an important bridge between controlled laboratory measurements and real-world gait monitoring.

Beyond their role as measurement tools, pressure-sensitive insoles have also been explored as therapeutic devices, exploiting the physiological principle of stochastic resonance to enhance somatosensory feedback during walking [156,157,158]. Stochastic resonance refers to the phenomenon in which the addition of low-amplitude, subsensory mechanical noise to a biological system can enhance its ability to detect weak signals, in this case, plantar mechanoreceptor inputs that underpin postural and locomotor control [159]. In practice, insoles embedded with piezoelectric or vibratory actuators deliver imperceptible broadband mechanical vibrations, set below each individual’s sensory threshold, to the plantar surface of the foot while standing and walking. The rationale is that this artificially augmented afferent signal helps compensate for the diminished somatosensory input that is characteristic of peripheral neuropathy and other neurological conditions. The foundational clinical evidence comes from a landmark study demonstrating that subsensory vibratory noise applied via shoe insoles improved postural balance and reduced gait variability in patients with diabetic neuropathy and stroke, two conditions in which plantar sensation is substantially reduced [159].

More recently, a randomized crossover trial confirmed that vibrating insoles acutely improved gait speed during level walking and stair descent in patients with diabetic peripheral neuropathy, with benefits most pronounced when the entire plantar surface was stimulated [160]. While the long-term effects on fall prevention and gait recovery remain to be established in larger trials, and their application across the broader spectrum of neurological disorders, including PD, cerebellar ataxia, and multiple sclerosis, remains largely unexplored, vibratory noise insoles represent a compelling convergence of measurement technology and therapeutic innovation, with potential for integration into rehabilitation programs as wearable, non-invasive sensory augmentation devices.

### 3.6. Consumer Devices: Scalability and the Challenges of Validation

The widespread adoption of smartphones and wearable consumer electronics has further expanded the possibilities for gait monitoring. Modern smartphones and smartwatches contain built-in accelerometers and gyroscopes that can record body movement with minimal user effort [161,162]. Because these devices are widely available and relatively inexpensive, they offer unprecedented opportunities for large-scale mobility monitoring in both clinical and population settings. In neurological research, smartphone-based monitoring has already demonstrated potential for remote assessment of gait disturbances in disorders such as PD, multiple sclerosis, and hereditary ataxias [163]. These approaches may enable clinicians to remotely track disease progression and identify mobility changes that require medical attention.

Despite their promise, consumer devices also present important challenges. Variability in device placement, differences in body size and movement patterns, and inconsistent patterns of device use can all affect measurement accuracy [138,164]. Moreover, most consumer technologies have not yet undergone rigorous validation against established laboratory methods. In addition to technical limitations, issues related to data privacy, secure transmission of health information, and ethical considerations surrounding digital monitoring remain important barriers to widespread clinical implementation [165]. For these reasons, consumer devices currently complement rather than replace established gait analysis methodologies.

A particularly promising horizon at the intersection of consumer technology and clinical neuroscience is the integration of artificial intelligence (AI), specifically machine learning and deep learning [166,167], into smartphone applications designed for gait monitoring. The accelerometer and gyroscope signals generated passively during everyday walking contain rich information about locomotor patterns that the unaided eye cannot discern; AI algorithms are uniquely capable of extracting clinically relevant features from these high-dimensional, time-series data streams [168].

Bibliometric analysis of the rapidly expanding literature confirms that machine learning and gait analysis have become one of the four dominant research themes in AI-assisted neurological diagnosis, with annual publications in this domain nearly quadrupling between 2018 and 2024 [168]. Convolutional neural networks and long short-term memory architectures have already demonstrated high classification accuracy for distinguishing pathological gait signatures in conditions such as PD, amyotrophic lateral sclerosis, and Huntington’s disease from gait data acquired with consumer-grade inertial sensors, in some studies exceeding 90% accuracy [168].

Looking ahead, one can envisage AI-driven smartphone applications capable not only of detecting early gait abnormalities in at-risk individuals but also of providing personalized real-time feedback, flagging clinically significant deterioration, and generating structured reports for remote clinician review. Such applications could serve as continuous digital biomarker platforms, enabling population-scale surveillance of motor health in ways that traditional laboratory methods cannot [169]. While regulatory pathways for AI-based medical software applications remain under active development, and prospective clinical validation in well-characterized neurological cohorts is still needed, the convergence of ubiquitous consumer devices with increasingly powerful AI gait analysis represents one of the most scientifically compelling directions in the field of neurological gait research [168].

### 3.7. The Road Ahead: Gait Analysis in the Era of Precision Neurology

The rapid expansion of gait analysis technologies reflects a broader transformation in neurology [67]. Traditional laboratory-based biomechanics is gradually giving way to more patient-centered approaches that capture mobility in real-world environments. This shift has important implications for both research and clinical practice [67].

Increasingly, gait metrics are recognized not merely as functional descriptors but also as digital biomarkers that can provide early signals of neurological dysfunction [67]. Subtle changes in gait dynamics may precede overt clinical symptoms in several neurodegenerative disorders, raising the possibility that mobility monitoring could contribute to earlier diagnosis and more timely therapeutic intervention [85,132,170].

Realizing this potential will require overcoming several methodological challenges. Harmonized protocols, rigorous validation across populations, and disease-specific analytical frameworks will be essential [171]. Equally important will be integrating gait metrics with complementary biological measures, including neuroimaging, electrophysiology, and molecular biomarkers [172]. In the coming decade, the goal should not simply be to refine measurement techniques but to embed gait analysis within the broader framework of precision neurology. In this emerging paradigm, mobility metrics will serve as a window into brain network function, enabling clinicians and researchers to detect neurological disease earlier, monitor its progression more accurately, and develop interventions tailored to individual patients’ needs [36].

## 4. Gait Analysis for the Differential Diagnosis of Neurological Disorders

### 4.1. Digital Phenotypes and the Characterization of Disease

Quantitative gait analysis has transformed the study of locomotion in neurological disease by providing a precise and objective framework for describing abnormalities in human movement. Historically, clinicians relied primarily on visual observation to identify characteristic gait patterns associated with neurological disorders [7]. Experienced neurologists can often recognize distinctive walking patterns, such as the short, shuffling steps of PD, the broad-based, irregular strides of cerebellar ataxia, or the asymmetric gait associated with hemiparetic stroke [7]. While such bedside observations remain essential to clinical practice, modern analytical techniques now allow these patterns to be quantified with a level of precision that was previously unattainable [173].

Contemporary gait analysis uses spatial–temporal parameters, joint kinematics, and kinetic measurements to generate detailed descriptions of locomotor behavior [174]. These measurements enable clinicians and researchers to identify disease-specific locomotor signatures—sometimes called digital phenotypes—that reflect underlying neurological dysfunction. For example, Parkinsonian gait is typically characterized by reduced step length, decreased walking velocity, and increased stride variability, whereas cerebellar ataxia is characterized by irregular step timing and widened base of support [175]. In hemiparetic gait following stroke, asymmetry between limbs often dominates the locomotor pattern.

The ability to quantify these patterns has important implications for both research and clinical care. Digital gait phenotypes can now be measured reproducibly, compared across patient populations, and followed longitudinally [67]. As a result, they offer valuable tools for monitoring disease progression and evaluating responses to therapeutic interventions. In disorders such as PD and multiple sclerosis, quantitative gait metrics have increasingly been explored as potential biomarkers that capture subtle functional changes that may not be apparent during routine clinical assessment [36,44].

Nevertheless, interpreting gait parameters requires careful clinical judgment. Walking behavior rarely reflects a single pathological process. Instead, locomotor performance emerges from the interaction of multiple factors, including neurological impairment, musculoskeletal limitations, cardiovascular fitness, and age-related physiological changes [95]. A patient with PD, for example, may simultaneously experience joint stiffness, muscle weakness, sensory deficits, and cognitive impairment, all of which can influence gait dynamics [46,176]. Consequently, quantitative gait metrics should not be interpreted in isolation.

The most reliable approach integrates gait analysis into a broader clinical framework that includes detailed medical history, neurological examination, and complementary investigations, such as neuroimaging, neurophysiology, or biofluid biomarkers, when appropriate [172]. Through such multimodal integration, gait analysis can move beyond descriptive measurement to illuminate the underlying pathophysiological mechanisms that contribute to mobility impairment [10,47].

Despite its considerable diagnostic potential, gait analysis has important limitations in the differential diagnosis of neurological disorders. Perhaps the most fundamental problem is phenotypic overlaps: many gait abnormalities are not pathognomonic for any single condition but rather emerge across a broad spectrum of diagnoses. Patients in early disease stages may display only subtle or non-specific locomotor changes that do not yet conform to a recognizable disease-specific pattern [177]. Furthermore, individual gait signs, such as reduced step length or increased stride variability, carry their own differential diagnosis and may be produced by entirely different underlying pathologies [177]. This is especially problematic in the parkinsonian syndromes, where distinguishing PD from atypical parkinsonism, including multiple system atrophy and progressive supranuclear palsy, on gait data alone is challenging. Machine learning approaches applied to large gait datasets have shown promising discrimination between these conditions, but gait-based models alone have exhibited high sensitivity at the cost of limited specificity, particularly for progressive supranuclear palsy [178]. Even when individual gait metrics show statistically significant group differences, the magnitude of overlap between patient populations typically precludes their use as standalone diagnostic criteria [7]. Finally, quantitative gait analysis remains largely absent from routine clinical workflows, partly because of the complexity of data interpretation and the lack of agreed normative values and disease-specific thresholds across different measurement platforms [67]. These limitations reinforce the view that gait analysis is best understood as one component of a comprehensive diagnostic evaluation rather than as a primary diagnostic instrument. In this context, Table 3 summarizes representative neurological gait phenotypes, their measurable gait signatures, and the primary neural substrates implicated in each pattern.

To preserve the broad transdiagnostic scope while improving disease-specific depth, Supplementary Table S1 summarizes the major gait signatures, candidate digital mobility outcomes, measurement technologies, clinical applications, and translational limitations across the principal neurological conditions discussed in this review.

### 4.2. Gait as a Prodrome: Early Markers of Neurological Decline

An increasingly important insight emerging from recent research is that gait abnormalities may appear long before the classic symptoms of many neurological disorders become clinically evident [47]. Quantitative gait analysis has revealed subtle changes in locomotor control that can precede overt motor impairment, suggesting that mobility metrics may serve as early indicators of neurological vulnerability [180].

One of the most widely studied parameters is stride-to-stride variability, which reflects the consistency of successive steps during walking. Increased variability has been associated with impaired neural control of locomotion and has been shown to predict cognitive decline and progression of neurodegenerative disorders in several longitudinal studies [74]. Similarly, an increase in double-support time, the phase of walking during which both feet remain in contact with the ground, often reflects compensatory strategies adopted by individuals who experience instability or reduced confidence in their balance [181].

These changes have been observed across a range of neurological and age-related conditions, including PD, multiple sclerosis, and frailty syndromes in older adults [19,132,147]. Such findings suggest that gait disturbances are not merely consequences of advanced disease but may represent early manifestations of subtle network dysfunction within the brain.

A growing body of evidence challenges the long-held assumption that gait deterioration in older individuals simply reflects normal aging; rather, such changes may signal the early stages of underlying neurological disease that has not yet become clinically manifest [182]. This conceptual reframing positions gait monitoring not merely as a tool for characterizing established disorders but as a proactive strategy for detecting prodromal neurological dysfunction, an approach that aligns with the broader ambitions of preventive neurology [182].

This perspective invites a broader conceptual shift in how gait is understood. Rather than viewing walking solely as a motor behavior governed by spinal and brainstem circuits, gait can be seen as a complex integrative function that reflects the coordinated activity of multiple neural systems [93]. Sensory processing, motor planning, executive function, and musculoskeletal mechanics all contribute to locomotion. Consequently, disturbances in gait may provide valuable insights into overall brain health.

The clinical implications of this concept are profound. If subtle changes in gait can identify individuals at increased risk of neurological decline, quantitative mobility monitoring could enable earlier diagnosis and intervention. Such an approach aligns with the growing emphasis on preventive neurology, in which disease detection occurs before irreversible damage to neural systems.

Despite the promise of gait as a prodromal marker, this concept carries important limitations that warrant careful consideration. Perhaps most critically, gait disturbances in older individuals are inherently multifactorial: musculoskeletal conditions such as osteoarthritis and chronic low back pain; peripheral neuropathy; medication side effects, particularly from sedatives, antidepressants, and antihypertensives; and anxiety related to fear of falling can all produce locomotor changes that closely resemble early neurological signs without any underlying neurodegenerative process [183,184]. This diagnostic confounding severely limits the positive predictive value of subtle gait changes when assessed in isolation and represents a significant obstacle to translating prodromal gait monitoring into clinical practice. Furthermore, many studies supporting gait as an early neurological biomarker have been cross-sectional or relied on relatively short follow-up periods, and a recent systematic review concluded that larger longitudinal datasets with appropriate adjustment for major confounders, particularly age, are still needed before gait measures can be validated for clinical use [185]. Gait variability, although sensitive across multiple neurological contexts, has been shown to carry low specificity as a standalone biomarker, limiting its utility for individual-level prediction [132]. Additionally, the absence of standardized gait testing protocols and universally agreed disease-specific thresholds further complicates the interpretation of prodromal signals across different platforms and populations [7]. These considerations do not diminish the conceptual importance of gait as a window into brain health, but they do reinforce that prodromal gait changes must be interpreted within a multimodal clinical context rather than as sufficient evidence of incipient neurological disease [182].

### 4.3. Toward Precision Medicine in Neurology

Taken together, advances in gait analysis suggest that locomotor assessment is evolving from a descriptive clinical tool into a central component of modern neurological diagnostics. Quantitative gait metrics are increasingly recognized as potential biomarkers that can capture the functional consequences of neurological disease with high sensitivity [86].

Future progress will depend on integrating gait analysis with complementary investigative modalities. Neuroimaging techniques such as structural and functional magnetic resonance imaging (MRI) can reveal alterations in brain networks that regulate locomotion, while electrophysiological methods provide insight into neural signal transmission and motor control [186]. Molecular biomarkers measured in blood or cerebrospinal fluid may provide additional insights into underlying neurodegenerative processes [187]. When combined with quantitative gait metrics, these approaches can provide a multidimensional picture of neurological disease.

Such integration supports the emerging paradigm of precision medicine in neurology, in which objective biomarkers guide early detection, enable patient stratification for targeted therapies, and provide sensitive outcome measures for clinical trials [86]. In disorders such as PD, where disease-modifying therapies remain an urgent unmet need, sensitive functional biomarkers could dramatically improve the evaluation of novel therapeutic strategies.

Despite these promising developments, several challenges must be addressed before digital gait phenotypes can be fully integrated into clinical practice. Methodological heterogeneity remains a major obstacle [188]. Different studies often employ distinct sensor technologies, analytical algorithms, and experimental protocols, limiting comparability across research centers [189]. In addition, many gait metrics have not yet undergone rigorous validation across diverse patient populations.

Overcoming these barriers will require coordinated international efforts to establish harmonized standards for gait measurement and analysis. Disease-specific calibration of gait metrics, large-scale longitudinal studies, and cross-platform reproducibility will be essential steps toward achieving robust clinical validation [190]. Only through such efforts can digital gait signatures evolve from research tools into reliable clinical biomarkers that support the next generation of precision neurology.

Yet even as harmonization efforts advance, it is important to recognize that the transition from research-grade gait analysis to validated clinical biomarker status remains a formidable challenge. Most gait metrics currently described in the literature have been validated in controlled laboratory or short-term clinical settings, and their performance in unstructured real-world environments, where walking conditions vary and medical comorbidities confound, is considerably less well established [188]. Furthermore, the field faces a reproducibility problem: algorithmic pipelines for computing gait parameters from raw sensor data differ substantially across research groups, meaning that a given metric may represent subtly different physiological phenomena depending on how it was calculated [132]. Clinical adoption will also require evidence that gait biomarkers add incremental diagnostic or prognostic value beyond that of standard clinical assessment, neuroimaging, and molecular biomarkers, a level of evidence that has not yet been rigorously demonstrated in large prospective trials [7]. Finally, equity considerations deserve explicit attention: digital gait monitoring technologies are not uniformly accessible across healthcare settings or patient populations, and evidence from diverse ethnic, socioeconomic, and geographic groups remains sparse [186]. These cautionary notes do not undermine the transformative potential of digital gait phenotyping in precision neurology, but they underscore the importance of building the evidence base with the same rigor that is expected of any proposed clinical biomarker.

## 5. Implications for Clinicians and Researchers

### 5.1. Digital Mobility Outcomes: A New Clinical Currency

One of the most important developments in modern gait analysis is the emergence of DMOs as objective measures of locomotor performance [191]. DMOs refer to quantitative metrics derived from sensor-based gait analysis that capture aspects of walking and balance in an automated and rater-independent manner [191]. Unlike traditional clinical assessments, which often rely on observational scoring systems or short performance tests conducted in controlled clinical environments, DMOs provide continuous and objective measurements of mobility.

This expanding measurement infrastructure has enabled DMOs to emerge as candidate biomarkers and trial endpoints, particularly when spatiotemporal gait metrics are extracted from sensors under free-living conditions. The most commonly reported DMOs are real-world walking speed, step length, stride time, cadence, and step count from body-worn IMUs or pressure insoles [188,191,192]. Three-dimensional markerless motion capture and single inertial sensors now achieve sufficient accuracy for kinematic assessment in community and clinic settings [188,193]. Clinically, DMOs differentiate PD from healthy controls and distinguish supervised from real-world gait, providing locomotor profiles from thousands of walking bouts recorded over days or weeks [188,191]. As progression biomarkers, digital gait measures detect longitudinal change in early PD when clinical rating scales do not, potentially enabling smaller disease-modifying trials [194]. Large international consortia, Mobilise-D, the Ataxia Global Initiative, are building validation frameworks so that these measures can serve as accepted performance outcomes and regulatory trial endpoints across PD, ataxia, fracture recovery, and other conditions [179,188,195]. Within digital rehabilitation trials, sit-to-stand performance, dynamic balance, and composite mobility scores have been shown to mediate gains in mobility and physical activity, linking training content to functional outcomes and offering mechanistic insight into how digital interventions work [196]. Key gaps remain in standardized acquisition protocols, predictive validity across disease stages, and ecological validity benchmarks to ensure laboratory-derived algorithms perform reliably in free-living conditions [179,188,191,192]. Robust, phenotype-stratified trials are needed to transform DMOs from promising research tools into routine clinical instruments.

These digital metrics can serve multiple purposes in clinical neurology. They may function as diagnostic adjuncts to characterize disease-related motor dysfunction, as prognostic indicators to predict clinical outcomes such as fall risk or loss of independence, and as sensitive outcome measures in clinical trials evaluating therapeutic interventions [7]. Importantly, many DMOs are derived from wearable technologies that capture mobility in free-living environments rather than laboratory settings [191]. This capability enhances ecological validity, allowing clinicians and researchers to observe how neurological disorders affect everyday mobility rather than performance during brief clinical assessments.

The rapid adoption of DMOs in clinical research reflects their growing relevance. Between 2010 and 2020, the incorporation of digital mobility metrics into clinical trials involving neurological disorders increased substantially, with use rising by nearly 40% [36]. Remote capture of these outcomes has also contributed to the decentralization of clinical research. By enabling participants to be monitored at home, digital mobility technologies can expand trial accessibility, reduce logistical barriers to participation, and improve the feasibility of studies involving rare or geographically dispersed populations.

Despite their promise, several conditions must be met before DMOs can achieve meaningful clinical impact. The minimally clinically important difference for each parameter must be clearly defined within specific disease contexts. In other words, researchers must determine the degree of change in a gait metric that corresponds to a clinically meaningful improvement or deterioration in patient function [197]. Moreover, digital mobility parameters must demonstrate clear associations with outcomes that matter to patients and clinicians, including fall risk, the need for assistive devices, and the ability to maintain independent living.

Disease-specific interpretation is also essential. Certain gait parameters may be highly informative in one disorder but less relevant in another. For example, stride length variability is particularly sensitive in PD, reflecting impaired basal ganglia control of locomotion [198]. The same parameter may be less informative in conditions such as multiple sclerosis or stroke, where other aspects of gait impairment predominate [19,132,147]. Without careful disease-specific calibration, the potential value of digital mobility outcomes risks being diluted by methodological inconsistency.

While the promise of DMOs is considerable, their translation into routine clinical practice remains in its early stages. A critical, unresolved challenge is the lack of agreed-upon reference values and clinically validated thresholds for most gait parameters across different neurological conditions and patient subgroups, a gap that limits clinicians’ ability to interpret individual-level results with confidence [188]. Furthermore, existing evidence on the clinical utility of DMOs is heavily concentrated in PD, with substantially fewer validation data available in conditions such as multiple sclerosis, stroke, or ataxia [191]. Patient-level factors, including age, body habitus, medication status, and comorbid musculoskeletal conditions, can alter gait parameters in ways unrelated to the primary neurological disease, potentially introducing noise or bias into DMO-based assessments [7]. Finally, from a health equity perspective, the populations studied in DMO research to date have been predominantly from high-income, specialist academic centers, raising concerns about the generalizability of reference standards and threshold values to diverse real-world clinical populations [198]. Addressing these limitations will require prospective multicenter studies with diverse, representative populations and standardized measurement frameworks, a shared agenda for both clinical neurologists and biomedical engineers.

### 5.2. Methodological Challenges and the Need for Consensus

Although quantitative gait analysis has advanced rapidly, several methodological challenges continue to limit its broader clinical adoption. Much of the existing research has compared well-defined patient groups with healthy control populations [67]. While such comparisons are useful for demonstrating proof of principle, they offer limited insight into real-world clinical decision-making, where clinicians must distinguish between overlapping neurological syndromes and detect subtle changes that may precede overt disease [199].

Future research will therefore need to move beyond simple case–control designs. More clinically relevant questions include whether quantitative gait metrics can identify prodromal stages of neurological disease, predict the transition from risk states to manifest disorders, and distinguish between conditions with similar clinical presentations [67]. In addition, gait metrics must demonstrate sufficient sensitivity to track disease progression and detect therapeutic effects in longitudinal studies [10].

Achieving these goals requires careful methodological rigor. Contemporary gait research employs a wide array of measurement technologies, including wearable inertial sensors, smartphone accelerometers, pressure-sensitive walkways, and video-based motion capture systems [67]. These devices are often paired with proprietary algorithms and analytical pipelines developed within individual research groups. Such heterogeneity complicates comparisons across studies and raises concerns about reproducibility.

Robust validation is therefore essential. Each device and analytical algorithm must be assessed for sensitivity, specificity, accuracy, and reliability across different populations and clinical contexts [138]. Increasingly, researchers are exploring sensor-fusion approaches that combine multiple measurement modalities to provide a more comprehensive description of locomotor behavior [200]. By integrating data from accelerometers, gyroscopes, pressure sensors, and video-based tracking systems, these approaches may capture the complexity of gait more effectively than any single measurement technique alone [132,147].

Equally important is thoughtful study design. Different experimental paradigms address distinct physiological questions. Treadmill walking allows controlled measurement of locomotor mechanics but may not reflect natural walking behavior [201]. Dual-task paradigms, in which individuals walk while performing a cognitive task, can reveal the interaction between locomotion and executive function [202]. Self-paced walking in real-world environments provides ecological validity but introduces additional variability. Without consensus on disease-appropriate tasks and measurement protocols, comparisons across studies will remain difficult [203]. Developing standardized frameworks for gait analysis is, therefore, a critical priority for the field.

A further underappreciated source of variability in gait research is intra-individual fluctuation: in conditions such as PD and multiple sclerosis, motor performance can vary substantially across the day, with medication state, fatigue, and symptom fluctuation all influencing gait parameters independently of disease progression [132,204]. Methodological frameworks must therefore account for within-person variability when defining measurement windows and interpreting change over time.

### 5.3. Remote Monitoring and the Promise of Real-World Data

Perhaps the most transformative innovation in gait research has been the ability to monitor mobility continuously in naturalistic environments. Advances in wearable sensors and mobile technologies now allow gait data to be collected outside the laboratory, providing a window into how neurological disease affects daily functioning [205].

Remote monitoring can be implemented through several technological models. In some studies, research-grade wearable devices are deployed in patients’ homes to collect high-resolution locomotion data over extended periods [206]. In other cases, clinical-grade sensors are integrated with smartphone applications that transmit data to centralized research platforms [96,207]. Increasingly, studies are also exploring the use of participant-owned consumer devices, such as smartphones and smartwatches, which contain built-in accelerometers and gyroscopes that can measure movement [163].

Each of these approaches involves important trade-offs. Research-grade devices typically provide the highest measurement accuracy but require logistical support, device distribution, and technical expertise [208]. Consumer devices are widely available and seamlessly integrated into daily life, but they often lack the rigorous clinical validation required for research applications [208]. Furthermore, interpreting real-world gait data is inherently complex. Mobility patterns can be influenced by environmental conditions, footwear, terrain, assistive devices, and individual differences in body composition.

To ensure that remote monitoring yields clinically meaningful information, researchers must adopt strategies that contextualize mobility data. These strategies may include patient diaries, concurrent monitoring of physical activity, or analytical techniques that adjust for environmental variables [209]. Only through careful validation and contextualization can real-world gait monitoring move from an experimental research tool to a routine component of clinical care.

The widespread deployment of remote monitoring technologies also raises important considerations around data privacy, informed consent, and the regulatory framework governing health data collected outside clinical settings, challenges that are distinct from those encountered in laboratory-based research and that require prospective ethical planning [165]. Moreover, continuous passive monitoring can generate large volumes of data that lack the ecological context needed for meaningful interpretation; without structured analytical approaches that account for activity type and time of day, raw mobility data risk producing noise rather than actionable clinical insight [209].

### 5.4. Artificial Intelligence and the Expanding Analytic Frontier

The rapid expansion of AI and machine learning has further increased the analytical potential of gait data [208]. These computational approaches enable the automated extraction of gait parameters from complex datasets and allow researchers to identify patterns that may not be detectable through conventional statistical analysis [210].

Recent studies have demonstrated that machine learning algorithms can extract locomotor metrics directly from video recordings, detect pathological signatures such as freezing of gait in PD, and identify subtle motor abnormalities that may precede clinical diagnosis [7,21,36,132]. Such tools have the potential to support earlier disease detection, improve longitudinal monitoring, and eventually contribute to automated clinical decision-support systems.

However, the clinical translation of AI-based gait analysis remains constrained by several challenges. Algorithms are typically trained on specific datasets that may not represent the diversity of real-world patient populations [211]. As a result, models that perform well in one cohort may demonstrate reduced accuracy when applied to different populations. Ensuring robust external validation across diverse clinical settings will therefore be essential before these tools can be widely adopted [165,188,212].

In addition to technical considerations, ethical and legal issues surrounding digital health data are becoming increasingly important [212]. Gait patterns are now recognized as biometric identifiers, enabling their use to identify individuals [213]. Regulatory frameworks such as the Illinois Biometric Information Privacy Act have begun to classify gait data as identifiable personal information, requiring strict safeguards for data storage, security, and consent. Addressing these governance challenges will be critical for maintaining public trust and ensuring the responsible implementation of digital mobility technologies.

These concerns extend beyond conventional data protection into a broader conceptual field now referred to as “neurorights”, a proposed framework of human rights specifically designed to protect individuals from the potential misuse of technologies capable of accessing, decoding, or altering neurological data [214]. Although the neurorights discourse has primarily developed around direct neural interface technologies and brain–computer interfaces, its principles are directly relevant to any technology capable of inferring sensitive information about a person’s neurological status, including digital gait analysis. Gait signatures, when processed by AI algorithms, may reveal not only movement disorders but potentially also cognitive state, emotional affect, and disease susceptibility, raising the prospect of unintended disclosure of neurologically sensitive personal information [214]. Ienca and Andorno proposed four specific neurorights, mental privacy, mental integrity, psychological continuity, and cognitive liberty, as candidate additions to the international human rights framework, arguing that existing legal instruments are insufficient to address the unique vulnerabilities created by neurotechnology [215]. As digital gait analysis systems become more sophisticated and increasingly capable of inferring neurological diagnoses and prognoses from behavioral data, the field must proactively engage with these ethical and legal frameworks to ensure that technological progress does not outpace the governance structures needed to protect patients’ rights and autonomy.

### 5.5. A Discipline at a Crossroads

Gait analysis now stands at a pivotal moment in its development. What began as a specialized field within laboratory biomechanics has evolved into a multidisciplinary domain that connects digital health technologies, neuroscience, and clinical medicine [7,216]. For clinicians, quantitative gait metrics offer new tools for diagnosing neurological disorders, predicting disease trajectories, and evaluating treatment outcomes [7]. For neuroscientists, gait analysis offers a non-invasive window into the functioning of neural networks that coordinate movement, balance, and cognition [217].

For patients, these developments hold particular promise. Continuous mobility monitoring may enable earlier detection of neurological disease, more accurate tracking of functional decline, and the tailoring of rehabilitation strategies to individual mobility profiles [218].

Nevertheless, the field must address several critical challenges before these benefits can be fully realized. Harmonized methodological standards, rigorous validation across populations, and robust ethical governance frameworks will be essential [132]. Rigorous validation across diverse patient populations, age groups, and disease stages is equally necessary before digital gait metrics can be considered clinically reliable biomarkers [10]. Finally, robust ethical governance frameworks must be developed to ensure that the collection, storage, and use of continuous mobility data respect patient autonomy, privacy, and equity [165]. If these conditions are met, gait analysis is poised to move beyond its traditional role as an observational tool and emerge as a central component of precision neurology, linking brain function, behavior, and disease through objective measurement of human mobility.

## 6. Recent Advances and Applications of Gait Analysis in Neurological Diseases

### 6.1. Parkinson’s Disease and Atypical Parkinsonism: From Shuffling Steps to Digital Biomarkers

The study of gait in PD has evolved from descriptive clinical observation to a quantitative discipline capable of revealing subtle disturbances in locomotor control. Historically, neurologists characterized Parkinsonian gait through visual assessment, noting short, shuffling steps, reduced arm swing, and a progressive reduction in stride length that typifies the disorder [219]. Advances in sensor-based gait analysis now allow these clinical features to be measured with high precision. Spatiotemporal parameters such as stride length, cadence, and gait variability provide objective indicators of motor dysfunction, while measurements of anticipatory postural adjustments—reflected in the latency and amplitude of center-of-pressure shifts before movement initiation—offer insight into the neural mechanisms underlying impaired gait initiation in PD and related parkinsonian syndromes [7,21,36,132].

These quantitative markers can reveal abnormalities that are often imperceptible during routine clinical examination. Wearable IMUs have been particularly valuable in this regard, enabling continuous monitoring of locomotor dynamics during everyday activities [88]. Because these devices capture movement in real-world environments, they provide a more comprehensive representation of functional mobility than laboratory-based assessments alone.

Sensor-derived gait metrics have also improved the ability to distinguish PD from atypical parkinsonian disorders. Differentiating these syndromes early in the disease course remains a major challenge in clinical neurology [220]. In progressive supranuclear palsy, for example, gait disturbances typically emerge earlier and are characterized by more severe postural instability and more frequent freezing episodes than in PD [221]. Quantitative gait analysis has identified objective locomotor signatures that help differentiate these disorders, thereby improving diagnostic accuracy [132,147]. Similarly, in normal-pressure hydrocephalus, gait parameters such as stride length and cadence show measurable improvement following cerebrospinal fluid diversion procedures, providing digital indicators of treatment response [222].

The incorporation of digital gait metrics into clinical trials further illustrates their growing relevance. In the PASADENA trial, which evaluated prasinezumab, a monoclonal antibody targeting α-synuclein, wearable sensor–derived gait measures were included as secondary outcomes and demonstrated greater sensitivity to functional change than conventional clinical rating scales [223]. Similar approaches have been applied in trials investigating metabolic interventions, such as ursodeoxycholic acid, in which subtle treatment-related changes in gait dynamics were detectable before differences became apparent on standard clinical scales.

Technological innovation continues to expand the possibilities for mobility monitoring in PD. Non-contact sensing technologies, including radiofrequency-based systems installed in patients’ homes, have recently demonstrated the ability to track fluctuations in gait velocity corresponding to levodopa responsiveness, thereby enabling continuous assessment of motor function in real-world settings [112].

Among the most disabling manifestations of advanced PD is freezing of gait, a phenomenon characterized by transient episodes in which patients are unable to initiate or continue walking. Electromyographic studies have identified characteristic tremulous muscle activity in the beta frequency range during freezing episodes, a signal summarized in the so-called freezing index [9]. More recently, machine learning algorithms applied to IMU and video datasets have enabled automated detection of freezing events with increasing accuracy. Recognizing the growing importance of these approaches, the 2023 International Workshop on Freezing of Gait recommended multisensor monitoring protocols that combine wearable IMUs with video-based analysis, while emphasizing the urgent need for harmonized analytic standards across studies [132]. Such advances point toward a future in which freezing episodes can be detected and continuously monitored, enabling personalized therapeutic adjustments and potentially adaptive neuromodulation strategies [224].

Beyond measurement, gait analysis is increasingly informing rehabilitation and technology-enhanced interventions in PD. Virtual reality (VR) gait and balance training improves step and stride length, balance, mobility, and quality of life relative to conventional therapy [225], while action observation and motor imagery, leveraging the mirror-neuron system, produce gains in gait, balance, disease severity, and quality of life [226,227,228]. Non-invasive brain stimulation, including repetitive transcranial magnetic stimulation (rTMS) and transcranial direct current stimulation (tDCS), shows mixed but promising effects; combining it with exercise improves Timed Up and Go, stride length, and UPDRS part III scores, though optimal stimulation parameters remain unsettled [229,230]. A particularly notable advance is closed-loop transcranial electrical stimulation synchronized to the gait cycle. Targeting the cerebellum, this approach increased gait speed, stride length, and symmetry while reducing subjective freezing in a randomized trial [231], exemplifying the move toward real-time, brain-state-dependent neuromodulation. A network meta-analysis of PD gait interventions found that aquatic therapy with dual-tasking and strength/balance training were among the most effective, while no intervention clearly treated freezing of gait [232].

### 6.2. Ataxia: Defining Digital Biomarkers of Cerebellar Dysfunction

Ataxic gait has long been recognized as a defining feature of cerebellar disease. Clinically, it is characterized by a widened base of support, irregular step timing, excessive stride-length variability, and pronounced truncal instability [233]. While these features can be recognized during bedside examination, quantitative gait analysis now enables their systematic measurement and links them to the underlying cerebellar dysfunction.

Recent efforts led by the Ataxia Global Initiative have sought to identify digital mobility parameters that best reflect disease severity in cerebellar disorders [179,234]. By comparing gait metrics with established clinical rating scales such as the Scale for the Assessment and Rating of Ataxia (SARA), investigators have identified several parameters that consistently correlate with disease burden [235]. These include gait velocity, measures of postural sway, stride-to-stride variability, and trunk range of motion during locomotion [236]. The identification of these parameters represents an important step toward standardizing digital biomarkers for cerebellar disease.

Advances in mobile technology have further expanded the possibilities for gait monitoring in ataxia. Smartphone-based systems can now capture multiple aspects of motor performance, including posture, tandem walking, and even speech-related motor coordination [86]. These digital measures are increasingly incorporated into multicenter research protocols to improve disease monitoring and evaluate novel therapies [236].

The potential of digital gait analysis to enhance clinical trials has also become evident. A reanalysis of the Troriluzole trial in spinocerebellar ataxia using deep learning-based motion analysis revealed treatment-related improvements in tandem gait stability that were not detected by traditional clinical scales [237]. This finding underscores the superior sensitivity of digital mobility biomarkers in capturing subtle therapeutic effects [237]. Beyond intervention studies, gait analysis has also demonstrated the ability to differentiate between subtypes of hereditary ataxia and to detect early locomotor abnormalities in fragile X–associated tremor/ataxia syndrome, thereby extending its utility to both early diagnosis and longitudinal disease tracking [238,239].

Quantitative gait analysis has emerged as a valuable tool for differentiating PD from cerebellar disorders, a distinction that can be clinically challenging, particularly in early disease stages where overlapping features complicate bedside assessment. The two conditions produce distinct and measurable locomotor signatures. Parkinsonian gait is characterized by a narrow base of support, reduced stride length, shuffling steps, diminished arm swing, and proportional velocity-stride length coupling, a pattern consistent with impaired basal ganglia-mediated force scaling [175]. Cerebellar ataxia, by contrast, produces a wide-based gait with excessive stride-to-stride variability, irregular step timing, and disproportionate increases in stride length relative to velocity, reflecting the cerebellum’s central role in online error correction and locomotor coordination [175,233]. Critically, gait variability, the beat-to-beat fluctuation in step timing and length, constitutes a key differentiating biomarker: it is substantially greater in cerebellar ataxia than in PD, even when overall walking speed is comparably reduced [70]. Cluster analysis studies using full kinematic datasets have confirmed that increased step width, combined with increased gait variability, reliably separates cerebellar ataxia from both PD and spastic paraplegia, offering a data-driven framework for differential diagnosis [240]. Beyond spatiotemporal parameters, arm swing provides an additional discriminating feature: in PD, arm swing amplitude is reduced and asymmetric due to extrapyramidal rigidity, whereas in cerebellar ataxia it may be preserved or alternatively dysmetric, with increased variability of amplitude and peak velocity [233]. Taken together, these quantitative gait signatures provide objective, technology-enabled criteria that can complement clinical examination in the differential diagnosis of movement disorders and that may ultimately support earlier and more accurate disease classification.

### 6.3. Multiple Sclerosis: Moving Beyond Traditional Disability Scales

Mobility impairment is one of the most prominent and disabling manifestations of multiple sclerosis (MS). For decades, clinical assessment of disability in MS has relied heavily on the Expanded Disability Status Scale (EDSS), which places considerable emphasis on ambulatory function. Although widely used, the EDSS has important limitations, including its relatively coarse resolution and limited sensitivity to subtle functional changes [241,242,243].

Quantitative gait analysis has revealed locomotor abnormalities in MS that conventional disability scales may not detect. Spatiotemporal parameters such as gait velocity, stride length asymmetry, and stride variability, as well as kinematic measures of joint range of motion, have demonstrated greater sensitivity to early impairment than EDSS scores alone [244]. Postural sway during dual-task walking—when individuals walk while performing a cognitive task—has also emerged as a sensitive indicator of neurological dysfunction in MS [245].

These insights are increasingly influencing both research and clinical practice. Wearable sensors and motion capture systems have been incorporated into clinical trials evaluating pharmacological therapies, neuromodulation strategies, and rehabilitation interventions for MS [246]. Consensus recommendations now emphasize the importance of disease-relevant gait parameters, such as stride asymmetry and postural sway variability, to harmonize studies and facilitate regulatory acceptance of digital outcomes [247]. Through these developments, gait analysis is beginning to provide precision that has long been lacking in traditional disability assessments.

Despite the diagnostic promise of quantitative gait analysis across PD, cerebellar ataxia, and multiple sclerosis, a fundamental methodological challenge persists: the same gait parameters are frequently altered by factors unrelated to the primary neurological diagnosis. Age-related gait changes, including reduced walking speed, shorter stride length, and increased step time variability, closely mimic the early locomotor signatures of PD and multiple sclerosis, making it difficult to attribute subtle gait deterioration to disease rather than normal aging [6]. Musculoskeletal comorbidities, such as hip osteoarthritis or lumbar stenosis, can produce gait patterns that superficially resemble parkinsonian shuffling or ataxic instability, while polypharmacy, particularly the use of psychotropic agents, vestibular suppressants, or anti-epileptic drugs, can independently impair balance and step regularity [6,67]. In multiple sclerosis, fatigue, heat sensitivity, and fluctuating spasticity introduce day-to-day intra-individual variability that complicates the detection of true longitudinal decline [95]. Taken together, these confounders mean that no single gait parameter can be interpreted in isolation; valid clinical conclusions require integration of the locomotor profile within the full clinical context. Automating the differential classification of neurological gait types represents a logical response to this challenge and one that AI-based video analysis is increasingly positioned to address. Unlike wearable sensors, which require device placement and patient cooperation, video-based markerless motion capture systems can capture full-body kinematics from standard digital cameras, a potentially scalable and low-cost approach for clinical and community settings [234]. Recent deep learning studies have demonstrated that convolutional and recurrent neural network architectures applied to multi-view video gait data can classify individuals with PD and multiple sclerosis from healthy controls with high accuracy, leveraging spatiotemporal joint trajectories extracted via pose estimation algorithms [248]. Such systems have the capacity to learn the composite locomotor phenotypes that distinguish these disorders, including the narrow base and reduced arm swing of Parkinsonian gait, the wide-based step irregularity of cerebellar ataxia, and the asymmetric spastic pattern of MS, without the need for expert observer input or specialized equipment. However, critical barriers remain before such tools can be deployed clinically: training datasets remain small and disease-homogeneous; models are rarely validated across different ethnic groups or disease stages; and the influence of confounders such as age, comorbidity, and medication is rarely explicitly modeled [7,67]. Developing AI-based gait classification systems that are trained on diverse, well-characterized multi-diagnosis cohorts and that explicitly account for non-neurological confounders represents one of the most important technical frontiers in the field.

### 6.4. Neuromuscular and Peripheral Nerve Disorders: Quantifying Weakness and Compensation

In disorders affecting peripheral nerves and muscles, gait abnormalities arise primarily from weakness, sensory loss, and structural deformities rather than central motor dysfunction [125]. Quantitative gait analysis offers valuable insight into how these impairments alter locomotor mechanics.

By measuring joint motion, ground reaction forces, and muscle activation patterns, gait analysis can reveal compensatory strategies individuals adopt in response to neuromuscular deficits [125]. For example, altered pressure distribution beneath the foot has been used to identify patients with diabetic neuropathy at increased risk of pressure ulcers [249]. Similarly, wearable sensors that track stride counts and walking speed have been used to monitor disease progression in muscular dystrophy and other inherited neuromuscular disorders [250].

In amyotrophic lateral sclerosis (ALS), walking speed has emerged as a particularly informative marker of disease progression. Declining gait velocity has been associated with worsening motor impairment and can predict the transition to reliance on assistive walking devices [251]. When combined with measures such as dynamometry and quantitative muscle testing, gait metrics provide a comprehensive picture of neuromuscular function and functional prognosis.

Quantitative gait analysis has also established itself as an essential outcome measure for evaluating the effects of botulinum toxin type A (BoNT-A) injections in conditions characterized by focal spasticity and abnormal muscle overactivity. BoNT-A is widely used in the management of lower limb spasticity following stroke, cerebral palsy, and hereditary spastic paraplegia, where excessive muscle tone constrains joint range of motion and disrupts the biomechanical efficiency of walking [252]. Three-dimensional kinematic gait analysis performed before and after BoNT-A injection can objectively quantify treatment-induced changes in ankle dorsiflexion, knee extension during stance, and step length, parameters that reflect the resolution of spastic muscle overactivity and the restoration of more physiological locomotor mechanics [253]. Critically, gait analysis can reveal not only improvements but also the unmasking of underlying weakness that may follow spasticity reduction, providing clinically important information that subjective scales alone cannot capture [252]. In hereditary spastic paraplegia specifically, mobile digital gait analysis using wearable inertial sensors has recently demonstrated sensitivity to BoNT-A treatment effects at one and three months post-injection, and machine learning models applied to pre-treatment gait parameters have shown preliminary capacity to predict which patients are likely to respond, opening a pathway toward precision dosing and patient selection [252]. In the context of the broader neuromuscular gait literature, gait analysis therefore serves a dual role: characterizing the baseline locomotor deficit and providing an objective, continuous window into therapeutic response across the temporal arc of treatment.

### 6.5. Stroke: Digital Metrics Guiding Recovery and Rehabilitation

Stroke frequently results in profound alterations in locomotor mechanics. Post-stroke gait is typically characterized by limb asymmetry, restricted joint mobility, abnormal force generation, and impaired postural control [254]. Common features include reduced ankle dorsiflexion, leading to foot drop; compensatory hip hiking to clear the foot during the swing phase; and trunk instability, which compromises balance.

Quantitative gait analysis has played a central role in identifying these biomechanical deficits and guiding rehabilitation strategies [255]. By measuring spatiotemporal asymmetry, joint motion, and postural stability, clinicians can tailor rehabilitation programs to address specific functional impairments.

Recent clinical trials have demonstrated the potential of novel rehabilitation technologies. Immersive virtual reality–based training programs have shown greater improvements in dynamic balance compared with conventional treadmill-based therapy [256,257]. Similarly, robot-assisted gait training systems allow patients to perform high-intensity, repetitive walking exercises while receiving real-time feedback on performance [254,257,258,259]. In these studies, digital gait metrics serve not only as measures of impairment but also as surrogate endpoints that capture therapeutic benefit with greater sensitivity than traditional functional scales.

Beyond VR and robotic approaches, digitally enabled rehabilitation is increasingly being tested in stroke and mixed neurological populations. The AMOUNT trial, which combined VR games, activity monitors, and tablets with usual care, produced modest improvements in objective mobility [260], while mobile health (mHealth) gait applications show significant gains in speed, cadence, step length, and Timed Up and Go versus controls. However, evidence quality is still limited [261]. Machine-learning models applied to wearable accelerometer data can now estimate multidimensional ground reaction forces in real time, opening the door to continuous, field-based monitoring of impact loads and personalized gait-retraining feedback outside the laboratory [262,263]. Combined with external-cue and implicit-learning evidence, sensor-driven biofeedback represents a promising route toward scalable intervention in both sport and clinical rehabilitation. Personalized gait rehabilitation stratified by gait phenotype or sensor-derived profiles remains largely aspirational [264], but current multimodal strategies are producing modest-to-meaningful gains that justify continued investment.

**Figure 6:**
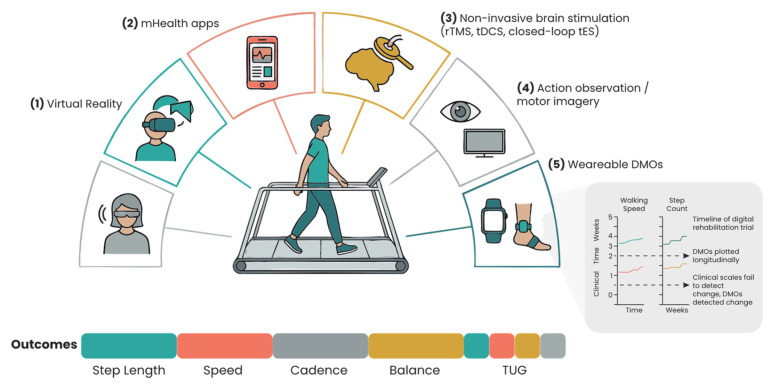
Technology-enhanced gait neurorehabilitation: interventions and digital outcomes. Schematic overview of digital, behavioral, and neuromodulatory approaches used in gait rehabilitation, including virtual reality, mHealth applications, non-invasive brain stimulation, action observation/motor imagery, and wearable-derived digital mobility outcomes. The lower bar summarizes common rehabilitation endpoints, and the right inset illustrates longitudinal tracking of wearable-derived gait measures during digital rehabilitation trials. DMO, digital mobility outcome; mHealth, mobile health; rTMS, repetitive transcranial magnetic stimulation; tDCS, transcranial direct current stimulation; tES, transcranial electrical stimulation; TUG, Timed Up and Go.

Outside controlled research environments, wearable inertial sensors are increasingly enabling continuous, real-world monitoring of gait recovery throughout the post-stroke rehabilitation continuum, from inpatient rehabilitation through community reintegration. Gait speed, one of the most robust and clinically interpretable post-stroke metrics, maps onto functionally meaningful ambulation categories: speeds below 0.4 m/s correspond to household-level ambulation, speeds between 0.4 and 0.8 m/s to limited community ambulation, and speeds above 0.8 m/s to full community independence, thresholds that carry direct implications for discharge planning and rehabilitation goal-setting [254]. Machine learning models trained on IMU data collected during brief in-clinic walking tasks have been shown to predict discharge ambulation status in post-stroke inpatient rehabilitation with greater accuracy than functional assessment scales alone, demonstrating that sensor-derived gait kinematics capture recovery-relevant information beyond what traditional clinical scores provide [265]. Extending this approach into more ecologically challenging environments, a recent 2026 study used machine learning analysis of trunk accelerometry to identify key gait stability features distinguishing stroke survivors from healthy controls during uneven-surface walking, revealing that even-surface gait speed below 0.8 m/s predicted significant outdoor instability and offering a framework for digital biomarker development targeting real-world community mobility [266]. In a complementary 2026 analysis applying supervised and unsupervised machine learning to five-point inertial sensor arrays, nine discriminative features spanning spatiotemporal, symmetry, stability, and smoothness domains correctly classified stroke patients from healthy controls with accuracies exceeding 94%, and the authors identified step symmetry and trunk harmonic ratio as the most diagnostically informative parameters, findings with direct implications for rehabilitation targeting [267,268]. Collectively, these advances position quantitative gait analysis not merely as a descriptor of post-stroke impairment but as a precision tool for longitudinal monitoring of recovery trajectories, prediction of functional outcomes, and personalized titration of rehabilitation intensity, a paradigm shift from episodic clinical measurement toward continuous, data-driven rehabilitation guidance (Figure 6).

### 6.6. Motor Learning, Orthotic Correction, and Biomechanical Gait Retraining

**Figure 7:**
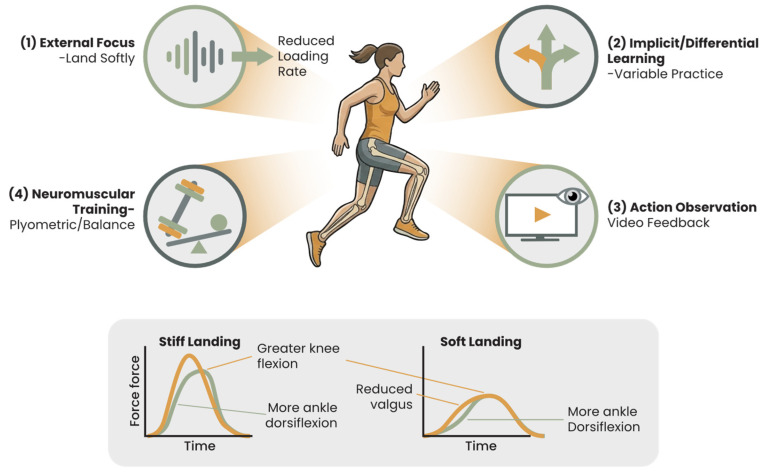
Motor learning principles for safer landing and gait retraining. Integrated framework showing four strategies used to modify lower-limb biomechanics during landing and gait-related tasks: external focus, implicit/differential learning, action observation with video feedback, and neuromuscular training. The lower panel contrasts stiff and soft landing patterns, illustrating differences in force-time profiles, knee flexion, ankle dorsiflexion, and dynamic knee valgus.

Gait analysis not only characterizes neurological impairment but also informs interventions designed to reshape locomotor mechanics through motor learning, feedback, and biomechanical assistance. How athletes learn to run and land safely is fundamentally a motor-learning problem: acquiring movement patterns that minimize injurious loading without degrading performance. High risk of anterior cruciate ligament (ACL) and ankle injury is consistently associated with “stiff” landing patterns, reduced knee and ankle flexion, elevated vGRF peaks, steep loading rates, and dynamic knee valgus [269,270,271]. Conversely, increased ankle dorsiflexion at initial contact improves energy dissipation and lowers peak vGRF and estimated ACL forces [271,272], while single-leg data confirm that more flexed, mobile strategies with controlled mediolateral and anteroposterior forces reduce joint loading and improve stability [273,274,275]. The most robust instructional finding is the superiority of external over internal attentional focus: cues such as “land softly and quietly” yield better mechanics, performance, and retention than body-focused instructions like “bend your knees more” [269,271,276,277,278]. In runners, simple “land soft” cues acutely reduce loading rates, vertical stiffness, and tibial acceleration without altering peak vGRF, effects comparable to barefoot or forefoot-strike instructions [278]. Implicit and differential learning approaches, in which high movement variability replaces prescriptive repetition, further promote automatic, stress-resistant patterns that are less susceptible to degradation under fatigue or competitive pressure [271,277,279]. Watching correct landings, expert videos, or one’s own feedback improves knee and hip flexion and can reduce dynamic knee valgus through mirror neuron engagement [269,270], and motivationally enriched environments, self-controlled practice, and contextual interference are proposed to capitalize on youth neuroplasticity for durable consolidation of injury-resistant mechanics [277,280,281,282]. Neuromuscular programs combining strength, balance, plyometrics, and jump stabilization improve landing control, reducing non-vertical forces and stabilization time, though adaptations decay without seasonal maintenance (Figure 7) [273,274].

**Figure 8:**
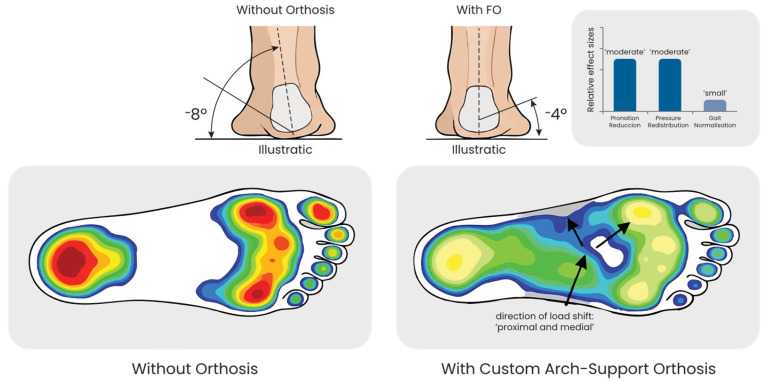
Foot orthosis mechanisms: plantar-pressure redistribution and rearfoot alignment. (**Top**) posterior views illustrate rearfoot alignment with and without foot orthosis support. (**Bottom**) plantar-pressure maps show redistribution of load after custom arch-support orthosis use, with reduced focal loading and a shift toward more proximal and medial regions. The inset summarizes relative effects on pronation reduction, pressure redistribution, and gait normalization. FO, foot orthosis.

Parallel to these sport-oriented strategies, foot orthoses (FOs) and insoles address pathological gait by passively modifying biomechanics. In flexible flatfoot, FOs reduce rearfoot eversion and inversion–eversion moments, though effects are small to moderate and evidence quality remains low [283,284,285,286]. Custom devices in plantar heel pain and rheumatoid arthritis reduce ankle eversion and plantarflexion moments [287,288,289,290], while in healthy runners, arch-support orthoses and medial posting reduce ankle inversion and tibial internal rotation [291,292]. The clearest and most consistent biomechanical action of FOs is plantar pressure redistribution: load shifts proximally and medially, unloading metatarsal heads and focal heel overload while increasing midfoot loading [283,289,290,291,293]. In flatfoot and plantar heel pain, custom orthoses reduce pathological motions, improve cadence and walking speed, and decrease pelvic-angle variability, collectively creating a more efficient, less aberrant gait pattern, though not a fully normal one [286,288,290,294,295]. In rheumatoid arthritis, orthoses reduce pain and ankle moments but do not alter daily step counts, suggesting symptomatic relief rather than a global gait overhaul [287]. In stroke and Charcot–Marie–Tooth disease, ankle–foot orthoses improve speed, cadence, and step/stride length [296,297]. Across tasks, however, foot orthosis effects concentrate at the ankle and midfoot; proximal mechanics remain largely unaffected [291,292], and the overall evidence for adult flatfoot is weak to moderate with few high-quality randomized trials (Figure 8) [283,284,286,294].

### 6.7. Emerging Directions Across Neurological Disorders

Across a wide range of neurological conditions—including PD, cerebellar ataxia, multiple sclerosis, peripheral neuropathy, ALS, and stroke—gait analysis is undergoing a profound transformation [70]. Advances in wearable sensors, artificial intelligence, and remote monitoring technologies have expanded the ability to continuously and objectively measure locomotor behavior.

This convergence of technologies is reshaping the clinical role of gait analysis. Locomotor metrics are increasingly used not only to describe functional impairment but also to inform diagnosis, predict disease trajectories, and evaluate therapeutic interventions [67]. The most promising developments lie in integrating gait analysis with complementary approaches, such as neuroimaging, electrophysiology, and molecular biomarkers [172]. Through such integration, locomotor behavior can be interpreted within the broader context of brain network function.

Recent research highlights the rapid progress occurring in this field. Over the coming decade, the central challenge will be to harmonize analytical methods, validate digital mobility metrics across diverse populations, and integrate these tools into routine clinical practice [188]. If these goals are achieved, gait analysis will move beyond its historical role as a descriptive tool and emerge as a cornerstone of diagnosis, prognosis, and therapeutic innovation in neurological disease.

The scale of this transformation is increasingly documented in the recent literature. A 2025 systematic review by Kirk et al. synthesized evidence from 27 studies examining real-world digital mobility outcomes in PD captured by wearable devices during everyday living, finding that walking speed, the most commonly reported measure, consistently distinguished patients from controls and real-world settings from supervised assessments but that between-study variability in devices, assessment protocols, and walking-bout definitions still limits direct comparison [191]. This finding exemplifies the central tension facing the field: sensor technology and analytical methods have outpaced the consensus infrastructure needed to make digital mobility outcomes interoperable and clinically actionable across conditions. In parallel, a large-scale Mobilise-D consortium study published in late 2025 assessed 24 technically validated digital mobility outcomes in over 600 people with PD monitored for seven days using a single wrist-worn device, confirming feasibility and compliance at scale while mapping mobility domains that differ systematically between patients and controls and across disease stages, a milestone in the translation of real-world gait metrics toward regulatory-grade endpoints [298]. Looking toward the full spectrum of neurological conditions, the integration of gait with complementary biological data streams is gaining pace. A 2025 narrative review established that upper-body gait signatures, such as arm swing asymmetry and trunk motion, serve as surrogate markers of disease susceptibility, pace parameters track progression, and gait variability, though sensitive, remains insufficiently specific to serve as a standalone biomarker, reinforcing the case for multimodal fusion [7,194]. Wearable technologies for gait rehabilitation have also seen a step change, with exoskeletons, functional electrical stimulation platforms, and AI-integrated biofeedback systems now combining real-time kinematic feedback with adaptive control, a development reviewed by Bartloff et al., who conclude that AI integration will define the next five years of neurorehabilitation by enabling data-driven, personalized therapy both in clinic and at home [299]. Alongside technical advances, the standardization challenge is receiving renewed attention: a 2026 systematic review of wearable sensors across clinical applications identified harmonized machine learning pipelines and integration with cloud-based health systems as prerequisites for scalable clinical translation, underscoring that the infrastructure layer remains as important as the technology itself [171].

## 7. Discussion

Gait, once regarded as a peripheral reflection of locomotor integrity, is now recognized as a sensitive biomarker of systemic and brain health. Disturbances of gait represent some of the most disabling features of neurological disease, driving falls, fractures, institutionalization, and premature mortality [9]. Nevertheless, despite this central role, our clinical and research paradigms remain far behind the complexity of gait itself. To move forward, gait analysis must be reconceptualized as a multidimensional marker of brain function, rather than merely measuring steps and stride. Achieving this goal requires addressing a series of persistent challenges that have hindered progress.

One of the most fundamental obstacles is the lack of a harmonized methodology and robust external validation. Many studies demonstrating the diagnostic or prognostic utility of digital gait biomarkers have been conducted in single-center, controlled settings with relatively homogeneous cohorts, while validation in independent populations, institutions, devices, and real-world environments remains less common. Devices, sensor placement, analytic pipelines, walking-bout definitions, and reporting practices differ so profoundly across centers that findings are rarely comparable, slowing progress and undermining reproducibility [21,36,204]. The Mobilise-D consortium has explicitly identified cross-device and cross-population validation as a central prerequisite for making digital mobility outcomes clinically interoperable [188], and a systematic review of real-world digital mobility outcomes in PD confirmed that between-study variability in devices, protocols, and walking-bout definitions still prevents direct comparison across studies [132]. Equally problematic is the inadequate validation of devices and algorithms in the very populations for which they are intended. Too often, reliability is established in young, healthy adults rather than in patients with PD, ataxia, multiple sclerosis, stroke, or neuromuscular disorders, precisely where it matters most [47,112,247,251]. Without rigorous external validation across disease populations, devices, clinical settings, and real-world environments, gait metrics risk remaining academic curiosities rather than clinically deployable tools.

These methodological limitations directly affect generalizability. Digital gait algorithms may perform less reliably when applied to populations that differ from the cohorts in which they were developed. Training datasets frequently oversample selected ambulatory participants with moderate disease severity while underrepresenting women, very old adults, early-stage disease, advanced disease, ethnic minorities, patients with multimorbidity, individuals using assistive devices, and populations in low-resource settings. Research also remains disproportionately focused on elderly adults in high-income countries, leaving children with cerebral palsy, adults with inherited ataxias, and many underserved populations underrepresented [236]. As Joseph (2025) has documented in a broader analysis of algorithmic bias in public health AI [300], models trained on homogeneous datasets may underperform in underrepresented populations, with potentially serious consequences for healthcare equity. Without deliberate inclusivity, subgroup performance reporting, and external validation in diverse cohorts, the promise of gait analysis could become unevenly distributed.

Taken together, these limitations highlight a central translational gap: most digital gait metrics remain research-grade biomarkers rather than clinically validated endpoints accepted by regulatory agencies or routinely implemented in neurological practice.

Regulatory qualification remains another major translational bottleneck. The tension between technological capability and methodological consensus sharpened considerably in 2026. Regulatory agencies have now demonstrated that the path from research metric to approved clinical endpoint is long and exacting: the Stride Velocity 95th Centile (SV95C), representing the 5% fastest real-world strides captured by a wearable device over 180 h, required fourteen years of collaborative work across academia, industry, patient groups, and regulators before receiving EMA qualification as a primary endpoint in Duchenne muscular dystrophy trials, making it the first wearable-derived digital gait parameter to achieve full regulatory status [301,302]. This landmark, the only regulatory-approved real-world digital gait endpoint for any neurological condition to date, underscores both the achievability and the scale of the challenge for conditions such as PD, multiple sclerosis, stroke, cerebellar ataxia, and peripheral neuropathies, where comparable qualification programs remain limited or at early stages. A 2026 systematic review of regulatory and research evidence confirmed that real-world walking speed, despite being among the most widely studied digital outcomes, remains insufficiently validated across most neurological conditions to meet the evidentiary requirements of either the U.S. Food and Drug Administration (FDA) or the European Medicines Agency (EMA) [301,302]. The barriers are not purely technical: they include the disease-specificity of current validation frameworks, which require separate qualification for each condition, even when the underlying measurement methodology is similar. Until cross-condition frameworks are developed and adopted, the field faces the paradox of accumulating data without accumulating regulatory credibility.

Cost–benefit considerations are also underdeveloped. Research-grade wearable monitoring is resource-intensive, requiring device procurement, participant training, technical support, data storage, algorithmic processing, cybersecurity safeguards, and clinician time for interpretation. Consumer-grade devices offer scalability and lower costs, but many lack disease-specific validation, transparent algorithms, and regulatory-grade performance [208]. A rigorous health-economic framework for digital gait monitoring in neurological practice has not yet been established [171]. Future implementation studies should therefore determine whether digital gait monitoring improves clinically meaningful outcomes, such as diagnostic accuracy, fall prevention, rehabilitation targeting, treatment selection, trial efficiency, or reduced healthcare utilization, enough to justify its operational demands. Without such evidence, routine adoption is likely to remain limited to specialized centers and research environments.

The historical reliance on laboratory-based methods further compounds the problem. Motion capture and force plates provide precision, but they constrain gait to artificial environments, limiting its natural expression. In reality, locomotion is an ecological behavior, shaped not only by motor pathways but also by cognition, affect, and environment. Wearable sensors and in-home monitoring have now revealed how gait unfolds in free-living settings, demonstrating patterns invisible under laboratory conditions [303]. This ecological shift is essential, for gait cannot be fully understood when stripped from the contexts in which it naturally operates.

At the same time, outcome measures remain fragmented. Some studies prioritize spatiotemporal variability, others focus on joint kinetics, sway, or electromyography. However, clinical practice demands clarity: which parameters truly predict clinically meaningful outcomes such as falls, institutionalization, or disease progression? Evidence suggests that stride-to-stride variability can anticipate cognitive decline, double-support time predicts fall risk, and walking speed predicts mortality across neurological conditions [73]. The field now urgently needs consensus on which digital mobility outcomes are most relevant for diagnosis and prognosis.

These challenges are also statistical and analytical, not only technological. Intra-subject variability must be carefully distinguished from true disease progression or treatment response because gait parameters can fluctuate with fatigue, medication state, time of day, footwear, attention, and environmental context. Test–retest reliability, standard error of measurement, and longitudinal responsiveness, therefore, need to be established within each target population before change scores can be interpreted clinically [72,74]. Similarly, minimal clinically important differences are still lacking for many gait parameters and neurological conditions, making it difficult to determine whether statistically significant changes correspond to meaningful functional improvement [197]. Reliable estimation of variability and nonlinear metrics also depends on adequate data length, since short walking bouts may produce unstable estimates of the stride-time coefficient of variation, entropy, or related measures [82]. In machine-learning applications, additional risks arise from high-dimensional feature sets, small and homogeneous samples, overfitting, incomplete reporting of preprocessing pipelines, and insufficient external validation. Robust gait biomarker research, therefore, requires prespecified feature-selection strategies, appropriate regularization, independent test sets, prospective validation, and transparent reporting standards [83,115,204,304]. Without this analytical rigor, digital gait metrics may appear statistically promising while remaining insufficiently reliable, responsive, or generalizable for clinical decision-making.

The scarcity of longitudinal data also limits progress. Cross-sectional studies cannot capture trajectories of decline or the transitions from prodromal to clinical syndromes. By contrast, prospective monitoring in Parkinson’s cohorts has revealed dynamic trajectories of freezing and instability that could never be appreciated from cross-sectional snapshots [132]. Without long-term, multicenter data, the field cannot progress from description to prediction.

Another barrier is the problem of specificity. Slow gait, a broad base, or a shortened stride length may reflect PD, cerebellar ataxia, or vascular cognitive impairment. Moving beyond descriptive overlap will require linking gait signatures to mechanistic circuits, where variability is anchored in cerebellar dysfunction, freezing is associated with fronto-striatal dysregulation, and cautious gait is linked to limbic–executive networks [305]. In parallel, integration with multimodal biomarkers remains underdeveloped. While neuroimaging, fluid biomarkers, and electrophysiology are advancing rapidly, they are rarely combined with gait metrics. Nevertheless, it is precisely this multimodal integration—already illustrated by studies linking gait variability to structural and functional MRI abnormalities—that holds the greatest promise for unlocking pathophysiological signatures [172].

The push toward multimodal integration is now extending to machine learning architectures that fuse gait data with complementary biological signals. A 2026 study in Current Opinion in Neurology demonstrated that combining positron emission tomography (PET)-derived tau staging with fluid biomarkers in Alzheimer’s disease and related disorders produces a richer staging model than either modality alone and explicitly proposed harmonized integration frameworks, accounting for age and sex, as prerequisites for translating multimodal data into prognostic tools [306]. Although focused on dementia biomarkers, this study articulates a principle directly applicable to gait research: that multimodal signals reflect related yet non-identical biological processes, and their fusion must be grounded in a rigorous understanding of what each modality independently measures before combining them. For gait in particular, this means that integrating wearable-derived locomotor metrics with neuroimaging, electrophysiology, or molecular biomarkers requires not only technical infrastructure but also conceptual clarity about what the gait signal is a proxy for, whether cerebellar error correction, fronto-striatal timing, or peripheral proprioceptive integrity, in order to avoid spurious correlations that obscure rather than illuminate pathophysiology [172].

Clinical translation is further hindered by the peripheral role gait metrics currently play in therapeutic trials. Even in landmark studies such as PASADENA, gait parameters have been included only as exploratory endpoints, lacking regulatory validation as surrogate endpoints [223]. Without systematic validation against hard outcomes such as falls, disability milestones, or mortality, digital mobility outcomes will remain on the margins of clinical trial design.

Machine-learning approaches introduce additional concerns related to bias, reproducibility, and interpretability. Model performance can be affected by selection bias, label noise, device heterogeneity, feature-engineering choices, preprocessing decisions, and incomplete reporting of hyperparameter optimization. Deep learning architectures, convolutional neural networks, recurrent networks, and transformer models can achieve high classification accuracy across neurological conditions but often function as black boxes, generating predictions that clinicians cannot interrogate or verify against clinical reasoning. This opacity directly limits clinical adoption: a model that correctly identifies early PD from gait dynamics but cannot indicate which features drove the prediction offers the neurologist little more than an unexplained probability score. Explainable AI (XAI) frameworks are now being systematically applied to gait analysis to address this gap. However, systematic reviews and recent applications of XAI to gait analysis have shown that different interpretability methods can identify different features as diagnostically important, and no standard currently exists for determining which features correspond to genuinely pathophysiologically meaningful signals [115,304]. A 2026 study published in Scientific Reports applied four distinct XAI methods (LIME, DeepLift, Integrated Gradients, and sequential feature selection) to deep learning models trained on three-dimensional gait kinematic data across multiple childhood gait disorders, demonstrating that different interpretability techniques identify different discriminative features and that no single method currently provides a reliable, reproducible picture of which gait parameters drive AI-based diagnostic classifications [304]. These findings highlight a critical methodological gap: the XAI literature for gait analysis remains fragmented, lacks standardization, and has not yet established whether the features identified as important by interpretability algorithms correspond to biomechanically or neurologically meaningful signals rather than to statistical artifacts of the training data. Until XAI outputs can be validated against established pathophysiological knowledge, and until their clinical meaningfulness is prospectively tested, the “interpretability” they appear to provide may offer false confidence rather than genuine transparency.

A further challenge lies in the neglect of cognitive and affective contributions to gait. Freezing, apraxia, and cautious gait are as much expressions of executive dysfunction and emotional modulation as they are of impaired motor circuitry [9]. Digital analysis must expand to capture these higher-order contributions, bridging gait research with the neuropsychology of attention, executive control, and mood.

Inclusivity and equity pose additional barriers. Research remains disproportionately focused on elderly adults in high-income countries, leaving children with cerebral palsy, adults with inherited ataxias, and populations in low-resource settings underrepresented [236]. (Moreover, technological inequities threaten to widen disparities: smartphones and wearables are not universally accessible, and algorithmic bias in machine learning models—trained on homogeneous datasets—risks propagating inequity in diagnosis and care [300]. Without rigorous external validation and deliberate inclusivity, the promise of gait analysis could become unevenly distributed.

The problem of inclusivity is compounded by a persistent focus on central motor disorders, principally PD and stroke, at the expense of neuromuscular conditions, which present distinct and underserved measurement challenges. In amyotrophic lateral sclerosis, Duchenne muscular dystrophy, hereditary spastic paraplegia, and spinal muscular atrophy, gait deterioration reflects peripheral motor unit loss, spasticity, and structural deformity rather than basal ganglia or cerebellar dysfunction, demanding biomarkers calibrated to these specific pathomechanisms [307].

Ethical and legal considerations add further complexity. As gait patterns are increasingly recognized as biometric identifiers, digital gait monitoring raises questions that extend beyond conventional data protection to include privacy, consent, data ownership, transparency, accountability, and equity. Passive wearable systems can generate pervasive behavioral data that extend beyond locomotor parameters, capturing activity rhythms, functional decline, social participation, and patterns of daily life in ways that participants may not fully anticipate. Consent frameworks must therefore address not only what data are collected but also who can access them, how long they are stored, whether participants can delete or retrieve their data, and whether secondary use for commercial, insurance, or other non-clinical purposes is prohibited. Regulatory instruments such as the General Data Protection Regulation and the EU AI Act provide relevant baseline protections, but their application to wearable health data collected outside conventional clinical settings remains complex and uneven. Data ownership is also unresolved, particularly when mobility data are generated through commercially operated platforms whose terms of service may grant broad rights to device manufacturers, creating tension with the principle that individuals should retain meaningful control over health-related data generated through their own daily activities. In this context, data portability, federated learning architectures, and transparent governance models may help reconcile algorithmic development with patient autonomy. AI-based gait analytics raise additional concerns regarding transparency, auditability, and accountability. Explainable AI methods are important, but they are not sufficient unless accompanied by institutional oversight, pre-specified performance monitoring, and clear audit trails that identify responsibility when algorithmic outputs contribute to diagnostic or therapeutic errors. The FDA guidance on Software as a Medical Device represents an important regulatory step toward lifecycle oversight of clinically deployed algorithms, but practical implementation remains challenging as models are updated, transferred across settings, or applied to populations different from those used for training. These concerns are inseparable from equity. AI systems trained predominantly on data from high-income, Western, and predominantly White populations consistently exhibit reduced performance when applied to underrepresented demographic groups, introducing a systematic diagnostic disadvantage for communities that already face disproportionate barriers to healthcare access. The cost of clinical-grade wearable devices, the connectivity requirements of remote monitoring platforms, and differences in digital literacy may further restrict access and compound existing disparities. Therefore, privacy protection, data ownership, algorithmic transparency, and equitable access must be treated as core dimensions of digital gait monitoring rather than as secondary considerations after technical validation [300].

Finally, clinical implementation requires more than technical validity. Most neurologists and rehabilitation specialists remain uncertain about how to interpret measures such as stride-time coefficient of variation, harmonic ratio, or center-of-mass stability margins. Digital gait reports are not yet integrated into standard electronic health record systems, and there is no consensus on how to communicate actionable findings to clinicians [173]. Without dedicated training, standardized reporting, clinical decision-support integration, and co-design involving clinicians, patients, health informatics specialists, and regulatory experts from the earliest stages of technology development, gait analysis risks remaining confined to the research arena [308].

The most pressing need, however, is conceptual. Gait is often reduced to locomotor mechanics when, in fact, it is a systems-level biomarker of brain and systemic health. The aspiration to treat gait alongside heart rate, blood pressure, temperature, respiration, and oxygen saturation captures this reconceptualization precisely: it demands not merely that gait be measured more frequently but that its clinical meaning be interpreted with the same urgency and sophistication applied to established physiological measures [86].

Four research priorities emerge as essential for the field to fulfill the potential of gait analysis as a cornerstone of precision neurology. The first is multimodal biomarker integration. Digital gait metrics will reach their full diagnostic and prognostic value only when combined with complementary biological signals, including neuroimaging measures such as MRI white matter burden and PET tau and alpha-synuclein staging, EEG, local field potential oscillations synchronized to gait events, and autonomic correlates of locomotion. Achieving this integration will require rigorous statistical models that explicitly account for cross-modality partial redundancy and biological specificity. The second priority is home-based and remote AI-assisted monitoring. The convergence of wearable devices with cloud-based analytics now makes continuous gait monitoring in everyday environments technically feasible at scale, but these systems must be externally validated, clinically interpretable, and integrated into care pathways before routine implementation. The third priority is the development of large-scale prospective longitudinal studies. Multi-year, multicenter cohorts spanning healthy aging, prodromal states, early disease, and advanced neurological disability are urgently needed to define trajectories of decline and determine which digital mobility outcomes predict clinically meaningful events. The fourth priority is the standardization of gait outcome measures. Coordinated international efforts, analogous to the work of the Mobilise-D consortium, are needed to produce disease-specific minimum datasets, algorithm benchmarks, reference ranges stratified by age, sex, and functional status, and consistent reporting standards. Regulatory agencies should be engaged from the earliest stages of this process to ensure that validation frameworks are built around clinically meaningful and pre-specified endpoints.

These priorities converge on a common translational objective: transforming gait analysis from a promising research tool into a clinically actionable framework for neurological assessment, prognosis, and therapeutic monitoring. Fortunately, this objective is attainable. The precedent set by the EMA qualification of SV95C for Duchenne muscular dystrophy demonstrates that, with sufficient multicenter collaboration, patient engagement, methodological harmonization, and regulatory dialogue, a wearable-derived gait parameter can meet the evidentiary standard required for drug development [301,302]. The task for the field is to extend this model across neurological diseases while ensuring that digital gait technologies are valid, interpretable, cost-effective, equitable, and ethically governed. In the coming decade, gait will not merely inform the neurological examination; it may continuously stream mobility intelligence from patients’ everyday environments to clinicians, researchers, and regulators, transforming a brief and episodic clinical encounter into a longitudinal window on brain health. Whether this transformation fulfills its promise will depend on whether the field can build methodological, economic, clinical, and ethical infrastructure equal to the technological opportunity now in reach.

## 8. Conclusions

Across neurological diseases, gait analysis has evolved from descriptive bedside observation to a quantitative discipline capable of informing diagnosis, prognosis, phenotyping, and therapeutic monitoring. Digital mobility outcomes provide rater-independent and ecologically valid measures that complement traditional clinical scales and may capture preclinical change, treatment response, and disease progression. However, their clinical translation remains incomplete. Methodological heterogeneity, limited external validation, inadequate representation of target populations, lack of consensus on disease-specific endpoints, and insufficient integration with other biological markers continue to limit routine implementation.

The next stage of the field should be defined by coordinated validation and clinical integration. Large, prospective, multicenter longitudinal studies are needed to determine which gait metrics reliably predict falls, disability progression, treatment response, institutionalization, and other clinically meaningful outcomes. Harmonized protocols, standardized reporting frameworks, and disease-specific reference ranges will be essential to improve reproducibility and comparability across devices, laboratories, and real-world settings. At the same time, the field should move beyond isolated spatiotemporal parameters toward disease-specific digital mobility phenotypes that integrate gait speed, variability, asymmetry, postural control, freezing, fatigability, turning, and dual-task performance according to the mechanisms and clinical priorities of each neurological disorder.

Future implementation will also require multimodal integration. Digital gait metrics should be combined with neuroimaging, EEG, electrophysiology, molecular markers, autonomic measures, and clinical outcomes to generate biologically interpretable models of locomotor dysfunction. Explainable AI frameworks will be necessary to transform high-dimensional gait data into transparent, auditable, and clinically meaningful outputs. Finally, ethical, regulatory, and health equity considerations must be incorporated from the outset, including data privacy, ownership of wearable-derived mobility data, algorithmic bias, accessibility, and cost-effectiveness. If these priorities are addressed, gait analysis can move from a promising research approach to a clinically actionable biomarker framework, standing alongside neuroimaging and molecular biomarkers as a cornerstone of precision neurology.

## Figures and Tables

**Table 1:** Core parameters in gait and balance analysis.

Domain	Parameter *	Definition and Clinical Relevance	Units
Spatial–Temporal	Velocity	Distance traveled per unit time →; global index of locomotor capacity; ↓ predicts frailty, mortality.	m/s, cm/s
	Stride length	Distance between same-foot heel strikes →; ↓ in bradykinesia, hemiparesis, ataxia.	m, cm
	Step length	Distance between contralateral footfalls →; asymmetry → hemiplegia, neuropathy.	m, cm
	Step/stride width	Lateral heel distance →; ↑ in cerebellar/sensory ataxia (mediolateral instability).	m, cm
	Cadence	Steps per minute, ↑ in PD as compensation for ↓ step length.	steps/min
	Variability	Step-to-step inconsistency ↔; ↑ signals impaired automaticity, ↑ fall risk, and cognitive decline.	—
	Single support time	Time on one limb; ↓ in bradykinesia, instability, limb pain.	s, %, min
	Double support time	Time when both feet contact the ground; ↑ in frailty and instability (strong fall-risk marker).	s, %, min
	Swing time	Time foot is in the air →; ↓ in PD, spastic gait.	s, %, min
	Stance time	Ground-contact duration: ↑ in cautious gait, ↓ in unstable gait.	s, %, min
Static Balance	Sway path length	COP excursion path →; ↑ in vestibular loss, sensory ataxia.	m, cm
	Sway velocity	Speed of COP/COM displacement →; ↑ predicts falls.	m/s
	Sway acceleration	Rate of sway velocity change →; ↑ indicates reduced responsiveness of postural control.	m/s^2^
	Sway area	COP trajectory area →; ↑ in MS and cerebellar disorders.	m^2^, cm^2^
	Sway amplitude	Max COP displacement →; ↑ with sensory deficits, neuropathy.	m, cm
	RMS sway	RMS sway amplitude ↔; ↑ when both amplitude and frequency components rise.	m/s^2^
	Jerk	Rate of change in acceleration (smoothness); ↑ = jerky, impaired postural corrections.	m^2^/s^5^
Dynamic Balance	Dynamic stability margin	xCOM distance from BOS →; ↓ in PD, ataxia, frailty →; ↑ fall risk.	m, cm
	Limit of stability	Max COP displacement tolerated beyond BOS →; ↓ indicates reduced functional reserve.	degrees
Kinematics	Joint angle	Segment orientation: abnormal ↑/↓ angles → spasticity, contractures, compensations.	degrees
	Joint motion	Joint excursion →; ↓ in PD, osteoarthritis, neuromuscular weakness.	degrees
Kinetics	Joint moment	Torque across the joint ⇧/⇩; reflects neuromuscular control and compensation.	Nm
	Force	Ground reaction or muscular force ⇧/⇩; ↓ in foot drop, weakness.	N
	Power	Torque × angular velocity; ↓ energy generation in aging, PD, myopathies.	W

* This set of parameters serves as the foundation for digital mobility outcomes in neurology. Spatiotemporal metrics capture the rhythm and scaling of locomotion, balance parameters reveal the integrity of postural control systems, and kinematic/kinetic measures quantify joint-specific contributions. Their integration not only enables precise phenotyping of disease-specific gait disturbances but also supports the development of digital biomarkers sensitive to preclinical changes, therapeutic effects, and long-term prognosis [7,36]. Abbreviations: PD = Parkinson’s disease; MS = multiple sclerosis; BOS = base of support; COM = center of mass; COP = center of pressure; N = newton; Nm = newton meter; RMS = root mean square; xCOM = extrapolated center of mass. Legend: → movement/distance; ↑ increased/prolonged; ↓ decreased/shortened; ↔ variability; ⇧ force/moment; ⇩ reduced force/power.

**Table 2:** Devices for gait analysis: methods, strengths, and limitations.

Device	Method *	Strengths (↑)	Limitations (↓)
Marker-based motion capture (mocap)	Infrared cameras track 3D marker trajectories → reconstruct segmental kinematics/kinetics.	↑ Gold standard for accuracy and reliability; ↑ detailed joint angles, forces, power; essential reference for validating new technologies.	↓ Requires laboratory environment; ↓ high cost; ↓ long setup time (2–3 h); ↔ marker displacement and soft-tissue artifact reduce accuracy.
Markerless motion capture	Pose-estimation algorithms extract joint centers and trajectories from RGB or depth video, without markers.	↑ Accessible and cost-effective; ↑ deployable in clinics and naturalistic settings; multi-camera integration ↑ accuracy; deep learning → improved localization.	Accuracy ↓ sensitive to lighting, clothing, and volume; ↓ less precise in frontal/transverse planes vs. mocap; ↔ lack of standardization across systems.
Pressure-sensitive walkways	Embedded pressure sensors → capture footfalls, producing spatiotemporal gait metrics.	↑ Accurate stride/step length, cadence, stance/swing times; ↑ validated in many neurological and musculoskeletal disorders; easy clinical use.	↓ Equipment bulky/expensive; ↓ not portable; cannot measure GRFs (⇧); ↓ not validated for long-term daily-life monitoring.
Force plates	Plates measure magnitude and direction of ground reaction forces (GRFs) → static and dynamic stability analysis.	↑ High-fidelity kinetic data (⇧); ↑ central tool in balance research; portable and embedded versions available.	↔ Inter-device variability reduces comparability; ↓ limited spatial–temporal data unless multiple plates are used; optimal accuracy in the lab only.
Wearable sensors (accelerometers, IMUs)	IMUs combine accelerometer + gyroscope + magnetometer → capture acceleration, angular velocity, orientation during real-world gait.	↑ Inexpensive, ↑ portable, ↑ scalable; ↑ real-world monitoring with high ecological validity; applicable across neurological populations.	Accuracy ↓ affected by drift, placement, interference, clothing, body habitus; ↔ large variability in algorithms; ↓ lack of harmonization reduces clinical adoption.
Smartwatches and smartphones	Embedded accelerometers and gyroscopes → track mobility continuously in daily life.	↑ Extremely scalable; ↑ integrates with digital health ecosystems; ↑ enables real-world monitoring and patient engagement.	↓ Reliability depends on device placement and user behavior; ↓ limited clinical validation; ↔ variability in devices worldwide; data privacy and security concerns.

* Each technology entails distinct trade-offs among precision, feasibility, and ecological validity. Marker-based mocap remains the benchmark for biomechanical fidelity but is impractical for routine clinical use. Markerless systems and pressure-sensitive walkways improve accessibility but struggle with real-world generalizability. Wearable sensors and consumer devices such as smartphones and smartwatches hold the greatest promise for scalability and ecological validity. Nevertheless, their adoption will require rigorous validation, harmonized analytic frameworks, and disease-specific optimization to ensure that digital mobility outcomes serve as reliable clinical endpoints [7,36,132]. Abbreviations: 3D = three-dimensional; GRF = ground reaction force; IMU = inertial measurement unit; mocap = motion capture; RGB = red–green–blue video. Legend: → method/process; ↑ advantage/increased precision; ↓ limitation/reduced accuracy; ⇧ force/kinetic measurement; ↔ variability issue.

**Table 3:** Neurological gait phenotypes, quantitative features, and neural substrates.

Gait Pattern *	Clinical Phenotype	Key Quantitative Gait Features	Primary Neural Substrate(s)
Typical parkinsonian gait	Bradykinesia → short, shuffling steps; ↓ foot clearance; ↓ heel strike; turning difficulty; postural instability progresses.	↓ velocity; ↓ step/stride length; narrow base; ↓ ROM; ↓ static/dynamic balance; ↑ cadence (compensation).	Basal ganglia (↓ dopaminergic drive), SMA (↓ internal cueing), and brainstem locomotor regions.
Atypical parkinsonism	Early, severe instability ↑; frequent early falls → PSP/MSA characteristics.	↓ velocity; ↓ step length; ↑ step width; ↑ double-support time; ↑ instability early on.	Basal ganglia–brainstem circuits; pedunculopontine nucleus; cerebellar involvement (MSA).
Ataxic gait	Broad-based, unsteady → irregular, staggering steps; difficulty with tandem gait.	↑ stride-to-stride variability ↔; ↑ step width; ↑ sway; ↓ static/dynamic stability.	Cerebellum (vermis, spinocerebellar tracts), vestibular nuclei, cortico-ponto-cerebellar pathways.
Hemiparetic gait	Unilateral weakness → foot drag, circumduction, spastic limb posture (typical stroke).	↑ asymmetry; ↓ ROM on affected side; abnormal kinematics; ↓ single-limb support; ↓ velocity/stride length.	Corticospinal tract damage (internal capsule, motor cortex); contralateral descending pathways.
Dystonic gait	Abnormal sustained leg postures → twisting, stiff gait; worsens with action/running.	sEMG: ↑ co-contraction (⇧ agonist–antagonist); abnormal kinematics; step asymmetry.	Basal ganglia (putamen, globus pallidus), sensorimotor cortex–basal ganglia circuits.
Spastic gait	Hypertonic, stiff-legged gait → scissoring, knee hyperextension, clonus.	↓ velocity; ↓ stride length; ↓ ROM; ↓ joint power; ↑ stance on unaffected side; ↓ force generation (⇩).	Corticospinal tracts; spinal interneurons; brainstem motor systems.
Neuromuscular disorders	Myopathy → Trendelenburg/waddling; Neuropathy/MND → foot drop (⇩ dorsiflexion) or steppage gait.	↓ velocity; ↓ stride length; ↓ ROM; ↓ power (⇩); abnormal EMG; ↑ balance instability; ↓ walking speed predicts prognosis (ALS).	Peripheral nerves (neuropathy), anterior horn cells (ALS/MND), NM junction, and muscle fibers (myopathy).
Functional gait disorders	Inconsistent, variable gait; marked distractibility; pattern ↑ inconsistent with organic disease.	↔ spatiotemporal inconsistency; improvement ↑ with distraction/dual-tasking.	No single structural correlate; dysfunctional top-down motor control → prefrontal, limbic, motor network integration issues.

* Neurological gait disorders present with characteristic clinical patterns that can now be objectively defined using digital analysis. Spatiotemporal parameters (e.g., velocity, step length, double support time) provide global measures of mobility. Kinematic and kinetic data reveal joint-level mechanisms, while EMG recordings uncover neuromuscular control and abnormal co-contraction. Emerging evidence underscores the diagnostic and prognostic value of gait variability, which predicts falls in ataxia [179]; double support time distinguishes atypical parkinsonism from PD [132]; and gait speed is associated with survival in older adults [8]. Functional gait disorders remain uniquely challenging, but quantitative tools offer new potential for distinguishing them from organic syndromes by capturing inconsistency and context dependence. Abbreviations: MND = motor neuron disease; MSA = multiple system atrophy; PSP = progressive supranuclear palsy; ALS = amyotrophic lateral sclerosis; SMA = supplementary motor area; NM = neuromuscular; ROM = range of motion; sEMG = surface electromyography. Legend: → characteristic feature; ↑ increased/exaggerated; ↓ decreased/reduced; ↔ variability; ⇧ excessive activation/force; ⇩ weakness or reduced activation.

## Data Availability

No new data were created or analyzed in this study. Data sharing is not applicable to this article. All graphs presented in the figures are schematic or representative illustrations and were not generated from original experimental or clinical data.

## Supplementary Materials

The following supporting information can be downloaded at: https://www.mdpi.com/article/10.3390/medsci14030338/s1, Table S1: Disease-specific gait biomarker summary across seven conditions.

## Abbreviations

The following abbreviations are used in this manuscript:

ACL	Anterior cruciate ligament
AD	Alzheimer’s disease
AI	Artificial intelligence
ALS	Amyotrophic lateral sclerosis
BoNT-A	Botulinum toxin type A
CE	Conformité Européenne/European conformity certification
CV	Coefficient of variation
DMO	Digital mobility outcome
EDSS	Expanded Disability Status Scale
EMA	European Medicines Agency
EMG	Electromyography
FDA	U.S. Food and Drug Administration
GRF	Ground reaction force
ICC	Intraclass correlation coefficient
IMU	Inertial measurement unit
LIME	Local Interpretable Model-Agnostic Explanations
mHealth	Mobile health
MRI	Magnetic resonance imaging
MS	Multiple sclerosis
PD	Parkinson’s disease
PET	Positron emission tomography
PKG	Personal KinetiGraph
rTMS	Repetitive transcranial magnetic stimulation
SV95C	Stride Velocity 95th Centile
tDCS	Transcranial direct current stimulation
tES	Transcranial electrical stimulation
TUG	Timed Up and Go
UPDRS	Unified Parkinson’s Disease Rating Scale
VR	Virtual reality
XAI	Explainable artificial intelligence
