# Supplementary material for: Walking as a Window to the Brain: Redefining Gait in Neurology
Source: Med Sci (Basel). 2026 Jun 23;14(3):338. doi: 10.3390/medsci14030338 (PMC13413934; doi:10.3390/medsci14030338)
Supplement: Supplementary file 1 [file medsci-14-00338-s001.zip › medsci-4342233-supplementary.pdf]

Table S1. Disease-specific gait biomarker summary across seven conditions.

Neurological condition	Major disease-specific gait signatures	Candidate digital mobility outcomes	Main measurement technologies	Potential clinical applications	Current translational limitations
Parkinson's disease [1-8]	Reduced gait speed and stride length; increased cadence as compensation for short steps; increased double-support time; reduced swing time; reduced hip excursion; reduced and asymmetric arm swing; impaired rhythmicity; increased stride-to-stride variability; freezing of gait; impaired turning and postural instability, especially in advanced disease.	Walking speed; stride/step length; cadence; double-support time; swing time; stride-time and step-length variability; arm swing amplitude and asymmetry; trunk regularity/smoothness; turning metrics; freezing-related indices; real-world walking-bout characteristics.	Instrumented walkways; pressure-sensitive mats; optical motion capture; wearable inertial measurement units placed on feet, trunk, waist, sternum, or wrists; smartphones and smartwatches; home-based and real-world monitoring platforms.	Early detection of subtle motor dysfunction; quantification of disease progression; fall-risk estimation; characterization of freezing of gait; monitoring medication, exercise, rehabilitation, and neuromodulation responses; candidate outcome measures for clinical trials.	Gait variability is sensitive but not disease-specific; medication state, fatigue, cognition, and environmental context strongly influence gait; protocols differ in sensor placement, walking-bout definition, task condition, and algorithm; real-world DMOs require further validation before routine regulatory use.
Atypical parkinsonism, including PSP and MSA [1,6,9-12]	Earlier and more severe postural instability than typical PD; early falls; widened base of support; increased double-support time; reduced gait speed and step length; impaired gait initiation and turning; lurching or unstable gait in PSP; cerebellar or autonomic-associated gait components in MSA; greater overlap with cerebellar and frontal gait patterns.	Double-support time; step width; gait speed; stride length; stride variability; postural sway; center-of-pressure displacement; turning instability; dynamic balance metrics; composite AI-derived gait/postural-instability classifiers.	Instrumented walkways such as GAITRite; posturography and pressure platforms; force plates; inertial sensors; multimodal gait-plus-postural-instability systems; machine-learning models combining gait and balance data.	Differential diagnosis between PD and atypical parkinsonian syndromes; early identification of PSP/MSA features; objective support for specialist referral; stratification in movement-disorder clinics; monitoring progression of axial disability.	Single gait parameters have substantial overlap between PD, PSP, MSA, and cerebellar ataxia; AI models may show high sensitivity but limited specificity unless gait and postural data are integrated; available studies often have small atypical-parkinsonism samples; external validation across centers remains limited.
Cerebellar ataxias [9,10,13-18]	Broad-based, unsteady, staggering gait; increased step width; irregular step timing; high stride-to-stride variability in spatial and temporal parameters; increased postural sway; impaired tandem gait; abnormal trunk control;	Stride-length variability; stride-time variability; step-width variability; gait speed; double-support time; trunk sway; trunk and arm-swing variability; postural sway area/velocity; tandem-walk metrics; SARA-correlated digital gait and balance markers.	Marker-based motion capture; electronic walkways; body-worn IMUs; trunk and limb sensors; camera-based or markerless systems; smartphone-based digital motor tests; real-world wearable monitoring.	Quantification of ataxia severity; detection of premanifest or early ataxia; monitoring disease progression; outcome measures for natural-history studies and clinical trials; differential diagnosis from PD, spastic paraplegia, vestibular	Rare-disease cohorts limit sample size and statistical power; protocols and metrics remain heterogeneous; gait speed strongly influences variability measures; longitudinal sensitivity and minimal clinically important differences remain incompletely defined; harmonized

	variable or dysmetric arm swing; impaired dynamic stability.			disorders, and functional gait disorders.	multicenter protocols are still needed.
Multiple sclerosis [3,4,7,14,19-22]	Reduced walking speed; shorter stride length; increased double-support time; impaired balance; increased fall risk; stride asymmetry; postural sway; fatigue-related gait deterioration; decreased hip extension during stance; decreased knee flexion during swing; decreased ankle dorsiflexion at initial contact and ankle plantarflexion during pre-swing.	Walking speed; stride length; cadence; double-support time; stride asymmetry; step-time variability; postural sway; dynamic balance metrics; walking-bout duration; step count; digital Timed Up and Go or 6-minute walk-derived metrics; patient-reported walking impact combined with sensor-derived gait.	Three-dimensional motion capture; instrumented walkways; wearable IMUs; lower-back or foot-worn sensors; smartphone-based mobility tests; real-world monitoring systems; clinical walking tests augmented with digital sensors.	Detection of subtle disability not captured by EDSS; monitoring functional mobility; fall-risk assessment; evaluation of rehabilitation or disease-modifying interventions; complementing patient-reported outcomes such as MSWS-12 and balance-confidence scales.	EDSS and clinical scales are relatively insensitive to subtle gait change; fatigue, heat sensitivity, relapses, medication, and day-to-day fluctuations confound interpretation; MS phenotype and disability stage influence gait metrics; real-world DMO validation and disease-specific thresholds remain incomplete.
Stroke [3,4,14,23-27]	Hemiparetic and asymmetric gait; reduced gait speed; shortened step length; reduced single-limb support on the paretic side; prolonged stance on the non-paretic side; foot drop; circumduction or hip hiking; reduced ankle dorsiflexion; abnormal joint moments and power; impaired balance and trunk control; increased temporal gait variability.	Gait speed; step-length asymmetry; stance/swing asymmetry; single-support time; paretic propulsion; joint angles; ankle dorsiflexion and foot-clearance metrics; trunk stability; cadence; stride-time variability; center-of-pressure/center-of-mass measures; digitally derived ambulation category.	Instrumented walkways; three-dimensional motion capture; force plates; wearable IMUs; foot pressure sensors; accelerometry; markerless video; mHealth platforms; machine-learning models for recovery or discharge prediction.	Characterizing gait impairment; rehabilitation planning; monitoring recovery trajectories; predicting community ambulation and discharge status; assessing response to treadmill, robotic, virtual-reality, mHealth, or sensor-based rehabilitation; stratifying patients by gait phenotype.	Stroke lesions and recovery stages are highly heterogeneous; compensatory gait strategies may mask impairment; musculoskeletal limitations, spasticity, weakness, cognition, and neglect confound gait metrics; wearable and AI approaches require broader validation in real-world rehabilitation settings.
Neuromuscular and peripheral nerve disorders [4,7,22,23,28]	Weakness-related slow gait; foot drop and steppage gait; sensory ataxia; widened base of support; reduced stride length and cadence; impaired ankle control; altered plantar pressure; reduced propulsion; increased postural instability; fatigue-related decline; in motor neuron or muscular	Walking speed; 2-minute/6-minute walk-derived distance and speed; stride length; cadence; foot-clearance and dorsiflexion metrics; plantar pressure distribution; stance/swing timing; gait ataxia and steppage severity scores; wearable-derived stride velocity, including SV95C in Duchenne muscular dystrophy;	Multi-sensor wearable systems; foot-worn IMUs; trunk sensors; pressure insoles; plantar-pressure platforms; digital biomechanical sensor arrays; real-world wearable monitoring; clinical walking tests augmented with sensors.	Objective grading of gait impairment; monitoring disease activity and progression; detecting clinically meaningful change over time; evaluating treatment response in inflammatory neuropathies, hereditary neuropathies, ALS, DMD, and spastic paraplegia; supporting regulatory-grade	Peripheral neuropathies and neuromuscular disorders are pathophysiologically heterogeneous; many clinical scales are subjective and insensitive to small changes; normative values and disease-specific MCIDs are limited; regulatory qualification is currently advanced mainly for selected outcomes such as SV95C in ambulant

	disorders, progressive loss of walking speed and endurance.	longitudinal change in gait biomechanics.		endpoints in selected neuromuscular diseases.	DMD, not across the whole neuromuscular spectrum.
Functional gait disorders [9,11,13,23]	Incongruous and inconsistent gait patterns; variability across time and context; marked distractibility; improvement with distraction or dual-tasking; excessive apparent instability without corresponding falls; bizarre or non-anatomical gait features; mismatch between observed impairment and preserved automatic movement capacity.	Within-session gait inconsistency; context-dependent variability; dual-task or distraction-related improvement; step-time and step-length irregularity; sway and balance metrics; discrepancy between supervised and unsupervised walking; pattern-recognition features distinguishing functional from organic gait disorders.	Standardized clinical video examination; instrumented walkways; wearable IMUs; posturography; markerless video analysis; automated pattern-recognition approaches; combined clinical-digital assessment protocols.	Supporting differential diagnosis from cerebellar, vestibular, parkinsonian, spastic, or neuropathic gait disorders; objective documentation of inconsistency and distractibility; monitoring response to physiotherapy or multidisciplinary rehabilitation; improving diagnostic confidence when integrated with clinical examination.	Functional gait disorders lack a single structural biomarker; digital evidence remains less developed than for PD, MS, stroke, or ataxia; algorithms may misclassify unusual organic gait patterns as functional; interpretation requires expert clinical context; standardized digital protocols for functional gait disorders remain underdeveloped.

Abbreviations: AI, artificial intelligence; ALS, amyotrophic lateral sclerosis; DMD, Duchenne muscular dystrophy; DMO, digital mobility outcome; EDSS, Expanded Disability Status Scale; IMU, inertial measurement unit; MCID, minimal clinically important difference; MSA, multiple system atrophy; MSWS-12, 12-item Multiple Sclerosis Walking Scale; PD, Parkinson's disease; PSP, progressive supranuclear palsy; SARA, Scale for the Assessment and Rating of Ataxia; SV95C, Stride Velocity 95th Centile.

1. Mirelman, A.; Bonato, P.; Camicioli, R.; Ellis, T.D.; Giladi, N.; Hamilton, J.L.; Hass, C.J.; Hausdorff, J.M.; Pelosin, E.; Almeida, Q.J. Gait impairments in Parkinson's disease. *Lancet Neurol.* **2019**, *18*, 697-708, doi:10.1016/S1474-4422(19)30044-4.
2. Zanardi, A.P.J.; da Silva, E.S.; Costa, R.R.; Passos-Monteiro, E.; Dos Santos, I.O.; Kruel, L.F.M.; Peyre-Tartaruga, L.A. Gait parameters of Parkinson's disease compared with healthy controls: a systematic review and meta-analysis. *Sci Rep* **2021**, *11*, 752, doi:10.1038/s41598-020-80768-2.
3. Warmerdam, E.; Hausdorff, J.M.; Atrsaei, A.; Zhou, Y.; Mirelman, A.; Aminian, K.; Espay, A.J.; Hansen, C.; Evers, L.J.W.; Keller, A., et al. Long-term unsupervised mobility assessment in movement disorders. *Lancet Neurol.* **2020**, *19*, 462-470, doi:10.1016/S1474-4422(19)30397-7.
4. Hulleck, A.; Mohan, D.M.; Abdallah, N.; Rich, M.E.; Khalaf, K. Present and future of gait assessment in clinical practice: Towards the application of novel trends and technologies. *Frontiers in Medical Technology* **2022**, *4*, doi:10.3389/fmedt.2022.901331.
5. Kalla, R.; Tiller, N.; Goyal, M.; Brémovà-Ertl, T. Gait as a vital sign: integrating wearables and AI into vestibular and balance medicine. *Front. Neurol.* **2026**, *17*, 1736898, doi:10.3389/fneur.2026.1736898.
6. Mancini, M.; Afshari, M.; Almeida, Q.; Amundsen-Huffmaster, S.; Balfany, K.; Camicioli, R.; Christiansen, C.; Dale, M.L.; Dibble, L.E.; Earhart, G.M., et al. Digital gait biomarkers in Parkinson's disease: susceptibility/risk, progression, response to exercise, and prognosis. *NPJ Parkinsons Dis* **2025**, *11*, 51, doi:10.1038/s41531-025-00897-1.
7. Micó-Amigo, M.; Bonci, T.; Paraschiv-Ionescu, A.; Ullrich, M.; Kirk, C.; Soltani, A.; Küderle, A.; Gazit, E.; Salis, F.; Alcock, L., et al. Assessing real-world gait with digital technology? Validation, insights and recommendations from the Mobilise-D consortium. *Journal of NeuroEngineering and Rehabilitation* **2023**, *20*, doi:10.1186/s12984-023-01198-5.
8. Kirk, C.; Packer, E.; Polhemus, A.; MacLean, M.K.; Bailey, H.; Kluge, F.; Gaßner, H.; Rochester, L.; Del Din, S.; Yarnall, A.J. A systematic review of real-world gait-related digital mobility outcomes in Parkinson's disease. *npj Digital Medicine* **2025**, *8*, 585, doi:10.1038/s41746-025-01938-y.
9. Fasano, A.; Bloem, B.R. Gait disorders. *Continuum (Minneap Minn)* **2013**, *19*, 1344-1382, doi:10.1212/01.CON.0000436159.33447.69.
10. Ebersbach, G.; Sojer, M.; Valldeoriola, F.; Wissel, J.; Muller, J.; Tolosa, E.; Poewe, W. Comparative analysis of gait in Parkinson's disease, cerebellar ataxia and subcortical arteriosclerotic encephalopathy. *Brain* **1999**, *122 (Pt 7)*, 1349-1355, doi:10.1093/brain/122.7.1349.

11. Nonnekes, J.; Goselink, R.J.M.; Růžička, E.; Fasano, A.; Nutt, J.G.; Bloem, B.R. Neurological disorders of gait, balance and posture: a sign-based approach. *Nat. Rev. Neurol.* **2018**, *14*, 183-189, doi:10.1038/nrneurol.2017.178.
12. Song, J.; Kim, J.; Lee, M.J.; Ahn, J.H.; Lee, D.Y.; Youn, J.; Chung, M.J.; Kim, Z.; Cho, J.W. Differential diagnosis between Parkinson's disease and atypical parkinsonism based on gait and postural instability: Artificial intelligence using an enhanced weight voting ensemble model. *Parkinsonism Relat. D.* **2022**, *98*, 32-37, doi:10.1016/j.parkreldis.2022.04.003.
13. Schniepp, R.; Möhwald, K.; Wuehr, M. Clinical and automated gait analysis in patients with vestibular, cerebellar, and functional gait disorders: perspectives and limitations. *J. Neurol.* **2019**, *266*, 118-122, doi:10.1007/s00415-019-09378-x.
14. Moon, Y.; Sung, J.; An, R.; Hernandez, M.E.; Sosnoff, J.J. Gait variability in people with neurological disorders: A systematic review and meta-analysis. *Human Movement Science* **2016**, *47*, 197-208, doi:10.1016/j.humov.2016.03.010.
15. Hausdorff, J.M. Gait variability: methods, modeling and meaning. *J Neuroeng Rehabil* **2005**, *2*, 19, doi:10.1186/1743-0003-2-19.
16. Ilg, W.; Milne, S.; Schmitz-Hübsch, T.; Alcock, L.; Beichert, L.; Bertini, E.; Mohamed Ibrahim, N.; Dawes, H.; Gomez, C.M.; Hanagasi, H., et al. Quantitative Gait and Balance Outcomes for Ataxia Trials: Consensus Recommendations by the Ataxia Global Initiative Working Group on Digital-Motor Biomarkers. *The Cerebellum* **2024**, *23*, 1566-1592, doi:10.1007/s12311-023-01625-2.
17. Kroneberg, D.; Numann, A.; Minnerop, M.; Ronnefarth, M.; Endres, M.; Kuhn, A.A.; Paul, F.; Doss, S.; Solbrig, S.; Elshehabi, M., et al. Gait Variability as a Potential Motor Marker of Cerebellar Disease-Relationship between Variability of Stride, Arm Swing and Trunk Movements, and Walking Speed. *Sensors (Basel)* **2024**, *24*, doi:10.3390/s24113476.
18. Ilg, W.; Muller, B.; Faber, J.; van Gaalen, J.; Hengel, H.; Vogt, I.R.; Hennes, G.; van de Warrenburg, B.; Klockgether, T.; Schols, L., et al. Digital Gait Biomarkers Allow to Capture 1-Year Longitudinal Change in Spinocerebellar Ataxia Type 3. *Mov Disord* **2022**, *37*, 2295-2301, doi:10.1002/mds.29206.
19. Lord, S.; Galna, B.; Verghese, J.; Coleman, S.; Burn, D.; Rochester, L. Independent Domains of Gait in Older Adults and Associated Motor and Nonmotor Attributes: Validation of a Factor Analysis Approach. *The Journals of Gerontology: Series A* **2013**, *68*, 820-827, doi:10.1093/gerona/gls255.
20. Sretenovic, I.; Potic, S.; Nedovic, G.; Odovic, G.; Simpraga, L. Functional Mobility Assessment in People with Multiple Sclerosis. *Neurol Int* **2025**, *17*, doi:10.3390/neurolint17050063.
21. Coca-Tapia, M.; Cuesta-Gomez, A.; Molina-Rueda, F.; Carratala-Tejada, M. Gait Pattern in People with Multiple Sclerosis: A Systematic Review. *Diagnostics (Basel)* **2021**, *11*, doi:10.3390/diagnostics11040584.
22. Poleur, M.; Tychon, C.; Gilbert, S.; Daumer, M.; Servais, L. Real-world walking speed as a digital biomarker and outcome measure for clinical trials—a systematic review, regulatory status and future directions. *Frontiers in Digital Health* **2026**, *8*, 1726549, doi:10.3389/fdgth.2026.1726549.
23. Ali, F.; Padilla, H.; Blazek, A.M.; Barnard, L.; Kaufman, K.R. Gait Analysis in Neurologic Disorders: Methodology, Applications, and Clinical Considerations. *Neurology* **2025**, *105*, e214154, doi:10.1212/WNL.0000000000214154.
24. Patel, P.; Enzastiga, D.; Casamento-Moran, A.; Christou, E.; Lodha, N. Increased temporal stride variability contributes to impaired gait coordination after stroke. *Sci. Rep.* **2022**, *12*, doi:10.1038/s41598-022-17017-1.
25. Koren, Y.; Barzel, O.; Shmuelof, L.; Handelzalts, S. Spatiotemporal variability after stroke reflects more than just slow walking velocity. *Gait & posture* **2024**, *110*, 59-64, doi:10.1016/j.gaitpost.2024.03.003.
26. Li, S.; Francisco, G.E.; Zhou, P. Post-stroke Hemiplegic Gait: New Perspective and Insights. *Frontiers in Physiology* **2018**, *9*, 1021, doi:10.3389/fphys.2018.01021.
27. Mohan, D.M.; Khandoker, A.H.; Wasti, S.A.; Ismail Ibrahim Ismail Alali, S.; Jelinek, H.F.; Khalaf, K. Assessment Methods of Post-stroke Gait: A Scoping Review of Technology-Driven Approaches to Gait Characterization and Analysis. *Front Neurol* **2021**, *12*, 650024, doi:10.3389/fneur.2021.650024.
28. Tejada-Illa, C.; Pegueroles, J.; Claramunt-Molet, M.; Pi-Cervera, A.; Heras-Delgado, A.; Gascón-Fontal, J.; Idelsohn-Zielonka, S.; Rico, M.; Vidal, N.; Martín-Aguilar, L., et al. Digital biomechanical assessment of gait in patients with peripheral neuropathies. *Journal of NeuroEngineering and Rehabilitation* **2025**, *22*, doi:10.1186/s12984-025-01694-w.
